# Comparative mitogenomic and evolutionary analysis of Lycaenidae (Insecta: Lepidoptera): Potential association with high-altitude adaptation

**DOI:** 10.3389/fgene.2023.1137588

**Published:** 2023-04-18

**Authors:** Wen-Ting Chen, Min Li, Shi-Yun Hu, Su-Hao Wang, Ming-Long Yuan

**Affiliations:** ^1^ State Key Laboratory of Herbage Improvement and Grassland Agro-Ecosystems, Lanzhou University, Lanzhou, Gansu, China; ^2^ Key Laboratory of Grassland Livestock Industry Innovation, Ministry of Agriculture and Rural Affairs, Lanzhou, Gansu, China; ^3^ College of Pastoral Agricultural Science and Technology, Lanzhou University, Lanzhou, Gansu, China; ^4^ National Demonstration Center for Experimental Grassland Science Education, Lanzhou University, Lanzhou, Gansu, China

**Keywords:** insects, Lycaenidae, comparative mitogenomics, phylogeny, high-altitude adaptation, non-coding regions

## Abstract

Harsh environments (e.g., hypoxia and cold temperatures) of the Qinghai–Tibetan Plateau have a substantial influence on adaptive evolution in various species. Some species in Lycaenidae, a large and widely distributed family of butterflies, are adapted to the Qinghai–Tibetan Plateau. Here, we sequenced four mitogenomes of two lycaenid species in the Qinghai–Tibetan Plateau and performed a detailed comparative mitogenomic analysis including nine other lycaenid mitogenomes (nine species) to explore the molecular basis of high-altitude adaptation. Based on mitogenomic data, Bayesian inference, and maximum likelihood methods, we recovered a lycaenid phylogeny of [Curetinae + (Aphnaeinae + (Lycaeninae + (Theclinae + Polyommatinae)))]. The gene content, gene arrangement, base composition, codon usage, and transfer RNA genes (sequence and structure) were highly conserved within Lycaenidae. *TrnS1* not only lacked the dihydrouridine arm but also showed anticodon and copy number diversity. The ratios of non-synonymous substitutions to synonymous substitutions of 13 protein-coding genes (PCGs) were less than 1.0, indicating that all PCGs evolved under purifying selection. However, signals of positive selection were detected in *cox1* in the two Qinghai–Tibetan Plateau lycaenid species, indicating that this gene may be associated with high-altitude adaptation. Three large non-coding regions, i.e., *rrnS*-*trnM* (control region), *trnQ-nad2*, and *trnS2-nad1*, were found in the mitogenomes of all lycaenid species. Conserved motifs in three non-coding regions (*trnE-trnF*, *trnS1-trnE*, and *trnP-nad6*) and long sequences in two non-coding regions (*nad6-cob* and *cob-trnS2*) were detected in the Qinghai-Tibetan Plateau lycaenid species, suggesting that these non-coding regions were involved in high-altitude adaptation. In addition to the characterization of Lycaenidae mitogenomes, this study highlights the importance of both PCGs and non-coding regions in high-altitude adaptation.

## 1 Introduction

Lycaenidae (Insecta: Lepidoptera: Papilionoidea), the second largest family of butterflies after Nymphalidae, consists of approximately 6,000 species in seven subfamilies (Pierce et al., 2002). Species in the family are widely distributed worldwide, with high diversity in morphology and ecology (Artem’eva, 2007; Schar et al., 2018). Lycaenidae species mainly inhabit mountains and forests, and some are adapted to high-altitude environments (Hughes, 2000; Balint et al., 2022; Marabuto et al., 2022). Most lycaenid species (about 75%) are associated with ants, forming a mutually beneficial symbiotic relationship (i.e., myrmecophily) (Pierce et al., 2002; Nemet et al., 2016; Riva et al., 2017; Kubik and Schorr, 2018), and this relationship may be related to the geographical distribution (Schmidt and Rice, 2002; Kaminski, 2008). However, little research has focused on the mechanism underlying environmental adaptation in Lycaenidae, and further analyses using molecular data are needed.

Mitochondria are the sites of energy conversion and metabolism in eukaryotes, known as the “energy factory” (Milenkovic et al., 2007; Tripodi et al., 2018; Bottje, 2019). Both nuclear and mitochondrial genomes (mitogenomes) encode essential proteins in the electron transfer chain of mitochondria. Generally, animal mitogenomes consist of 37 genes, i.e., 13 protein-coding genes (PCGs), 22 transfer RNA genes (tRNAs), and two ribosomal RNA unit genes (rRNAs, *rrnL* and *rrnS*). In addition, animal mitogenomes usually contain a large non-coding region, known as the control region (CR) or AT-rich region in arthropods, which contains essential regulatory elements for transcription and replication (Boore, 1999; Zardoya and Suadrez, 2008). Mitogenomes have been used in population genetics, phylogeography, and phylogenetic studies of various taxa, e.g., insects (Yuan et al., 2015; Liu et al., 2022a; Zhang et al., 2022), spiders (Tyagi et al., 2020; Li et al., 2021; Li et al., 2022), and centipedes (Hu et al., 2020; Ding et al., 2022). To date, only nine sequenced mitogenomes of nine Lycaenidae species have been stored in GenBank, which is extremely limited given the species richness, restricting our understanding of the phylogeny and evolution of the family.

It is historically believed that mitogenomes evolve neutrally; however, mitochondrial genes have important functional roles in OXPHOS, suggesting that they are targets of natural selection (Blier et al., 2001; Manoli et al., 2007; da Fonseca et al., 2008). Signals of adaptive evolution in several mitochondrial genes have been detected in various animal taxa, e.g., *atp6* in wild Tibetan pigs (Li et al., 2016), *atp8* in *Freyastera benthophila* (Mu et al., 2018) and Gobiidae (Shang et al., 2022), and *cob* in *Calyptogena marissinica* (Yang et al., 2019a). The Qinghai–Tibetan Plateau (QTP) is the largest plateau in the world, characterized by hypoxia, cold temperatures, and strong ultraviolet radiation. These harsh environmental conditions influence species diversification and adaptive evolution substantially (Zhang et al., 2017; Yuan et al., 2018). Non-neutral evolution in mitochondrial genes has been found in birds (Zhou et al., 2014; Gu et al., 2016), mammals (Yu et al., 2011; Luo et al., 2012; Peng et al., 2012), fish (Li et al., 2013; Wang et al., 2016), and insects (Zhang et al., 2017; Yuan et al., 2018; Balint et al., 2022) inhabiting the QTP. Further mitogenomic analyses of additional QTP insect groups will improve our understanding of adaptation to high-altitude environments. A large number of genetic analyses have shown that mitogenomes can be used to analyze adaptive evolution (Korkmaz et al., 2017; Yuan et al., 2018; Yuan et al., 2020; Bartakova et al., 2021).

In this study, we proposed that high-altitude adaptation in Lycaenidae inhabiting the QTP is associated not only with PCGs but also non-coding regions. We sequenced four complete mitogenomes of two QTP lycaenid species, *Polyommatus amorata* and *Agriades orbitulus*. Combined with sequenced mitogenomes of nine lycaenid species available on GenBank, we performed a detailed comparative mitogenomic analysis and constructed a mitogenomic phylogeny of Lycaenidae. We focused on the importance of PCGs and non-coding regions in the environmental adaptation of Lycaenidae. In addition to characterizing Lycaenidae mitogenomes, our results provide new insights into the high-altitude adaptation and evolution of Lycaenidae.

## 2 Materials and methods

### 2.1 Sampling, DNA extraction, and sequencing

Adult specimens were collected from alpine meadows of the QTP, in Menyuan County of Qinghai Province and Naqu County of the Tibet Autonomous Region, China. Detailed sampling information is provided in Supplementary Table S1. Samples were preserved in 100% ethanol during collection and stored at −80°C after transporting to the laboratory until DNA extraction. Samples were deposited in the State Key Laboratory of Herbage Improvement and Grassland Agro-Ecosystems, College of Pastoral Agricultural Science and Technology, Lanzhou University, Lanzhou, China. Total genomic DNA was extracted from a single specimen using a DNeasy Tissue Kit (Qiagen, Hilden, Germany). The DNA quality was detected by 1.2% agarose gel electrophoresis and spectrophotometry using the NanoDrop ND-1000 (Thermo Fisher Scientific, Waltham, MA, United States). DNA was sequenced in both directions using the Illumina NovaSeq 6000 platform (2 × 150 bp) by Wuhan Benagen Tech Solutions (Wuhan, China).

### 2.2 Mitochondrial genome assembly, annotation, and analysis

Low-quality reads, including reads with a cutoff Phred quality score of Q20, more than 5% N bases, adapter sequences, or repeated reads introduced by PCR duplicates were removed using SOAPnuke (version: 2.1.0) (Chen et al., 2017). The high-quality reads were assembled by using SPAdes (version 3.13.0) (Bankevich et al., 2012), using the mitogenome of *Cupido argiades* (NC_023088) as a reference. The assembled mitogenomes were annotated by the MITOS web server (http://mitos2.bioinf.uni-leipzig.de) (Bernt et al., 2013) to locate PCGs, tRNAs, and rRNAs by comparisons with homologous regions in other insect mitogenome sequences. All identified PCGs were corrected by sequence alignment using the published Lycaenidae mitogenome sequences, the start and stop codons were identified, and all genes were manually verified and proofread after annotation (to avoid overlap). The secondary structures of the tRNA genes were verified using tRNAscan-SE (version 1.21) (Lowe and Eddy, 1997). The tandem repeats of control regions were detected using Tandem Repeats Finder (version 4.09) (Benson, 1999). All four newly sequenced mitogenome sequences have been deposited at GenBank (under accession number ON411617-20).

Nucleotide diversity (π) was calculated for the 13 PCGs of the mitochondrial genome using DnaSP (version 6) with a sliding window of 100 bp and a step size of 25 bp (Rozas et al., 2017). The nucleotide composition and codon usage were analyzed using MEGA (version 10.2) (Kumar et al., 2018). The GC content, GC-skew, and AT-skew were estimated to evaluate the overall nucleotide composition. The GC content (GC %) was defined as the content of G + C, and the formula for compositional skewness was as follows: AT-skew = [A−T]/[A + T], GC-skew = [G−C]/[G + C] (Perna and Kocher, 1995). The effective number of codons (ENCs), codon bias index (CBI), and G + C contents of the first, second, and third codon positions were used to analyze codon usage. The ENC and CBI were determined using DnaSP (version 6) (Rozas et al., 2017), and the G + C contents at the first, second, and third codon positions were determined using MEGA (version 10.2) (Kumar et al., 2018). The correlation between the G + C content of all codons (GC_a_), G + C content of the third codon position (GC_3_), ENC, and CBI as well as the relationship between nucleotide composition and codon bias for all PCGs were analyzed using Excel. Secondary structures of sequences were predicted by the minimum free energy model using mfold (http://www.unafold.org/) (Zuker, 2003). The most stable structure (i.e., the structure with the lowest free energy) was selected when there were multiple structures.

### 2.3 Phylogenetic analysis

Thirteen lycaenid mitogenomes were included in a phylogenetic analysis, including the four newly sequenced mitogenomes and nine lycaenid mitogenomes (nine species) available from GenBank (Supplementary Table S2). *Apodemia mormo* (NC_024571) and *Abisara fylloides* (NC_021746) belonging to Riodinidae were used as outgroups. All 13 PCGs were individually aligned by ClustalW (Codons), and two rRNAs (*rrnL* and *rrnS*) were aligned by ClustalW, implemented in MEGA (version 10.2) (Kumar et al., 2018). Poorly aligned and divergent sequences were removed using the Gblocks server (http://molevol.cmima.csic.es/castresana/Gblocks_server.html). Three datasets were generated for phylogenetic analyses: 1) the P123 dataset, with nucleotide sequences at all codon positions of 13 PCGs; 2) the P123RNA dataset, with P123 and the nucleotide sequences of two rRNAs; 3) the P123AA dataset, with the inferred amino acid sequences of 13 PCGs. Each dataset was tested for substitution saturation by using DAMBE (version 5.3.74) (Xia, 2013). There was no substantial sequence saturation (Supplementary Table S3), indicating that the datasets can be used in phylogenetic analysis. The best partitioning schemes and corresponding nucleotide substitution models for each dataset were identified using the IQ-TREE web server (http://iqtree.cibiv.univie.ac.at/) (Trifinopoulos et al., 2016), and the results were used for downstream phylogenetic analysis (Supplementary Table S4).

Maximum likelihood (ML) phylogenetic analysis was performed using RAxML-HPC2 (version 8.0.24) (Stamatakis, 2014) with the GTRGAMMA model and 1,000 bootstrap (BS) replicates. Bayesian inference (BI) was performed using MrBayes (version 3.2.7) (Ronquist et al., 2012), with 1 × 10^8^ generations and sampling every 100 generations (Yuan et al., 2015). Stationarity was achieved when the estimated sample size was over 100 and when the potential scale reduction factor approached 1.0, and default settings were used for the remaining parameters (Ronquist et al., 2012).

### 2.4 Evolutionary rates and selective pressure analysis

The number of synonymous substitutions per synonymous site (*d*_S_), the number of non-synonymous substitutions per non-synonymous site (*d*_N_), and the ratio of non-synonymous to synonymous substitutions (*ω*) were calculated for the 13 PCGs in Lycaenidae. Generally, *ω* (*d*_N_/*d*_S_) < 1 indicates negative/purifying selection, *ω* > 1 indicates positive/diversifying selection, and *ω* = 1 indicates neutral expectation (Anisimova and Kosiol, 2009). All *d*_N_ and *d*_S_ values were calculated using MEGA (version 10.2) (Kumar et al., 2018), and *ω* was calculated using Excel. To analyze the selective pressure for each PCG in Polyommatinae species under branch-specific and branch-site models, the CodeML program in PAML (version 4.7) was used, applying a ML approach (Yang, 2007). Two Polyommatinae species (*P. amorata* and *A. orbitulus*) inhabiting the QTP were used as the foreground branch. Positive selection was inferred when *ω* > 1, and the log-likelihood ratio test (LRT) was significant (*p* < 0.1) (Yang, 2007). The Bayes empirical Bayes (BEB) method was used to calculate posterior probabilities for site classes to determine which codon positions experienced positive selection (*ω* > 1) (Zhang et al., 2005).

We also used the Datamonkey web server (http://www.datamonkey.org/) to analyze the evolutionary rate of each PCG, with the fixed-effects likelihood mode (FEL, site-by-site analysis) to detect which codons were under selection (Pond and Frost, 2005).

## 3 Results

### 3.1 General features of Lycaenidae mitogenomes

We obtained four complete mitogenomes for two QTP Polyommatinae species (Supplementary Table S1). These newly sequenced mitogenomes were closed circular DNA molecules, with sizes ranging from 15,340 bp (*A. orbitulus* NQ2) to 15,389 bp (*P. amorata*), similar to those of other Lycaenidae species (15,162–15,366 bp) (Supplementary Table S2). Thirty-seven typical mitochondrial genes without rearrangements were detected in each mitogenome, comprising 13 PCGs, 22 tRNAs, and two rRNAs. Non-coding regions, consisting of putative control regions (CRs) and 879 bp intergenic nucleotides dispersed among 68 intergenic regions (IGRs) in four newly sequenced mitogenomes, were similar in all lycaenid species.

**FIGURE 1:**
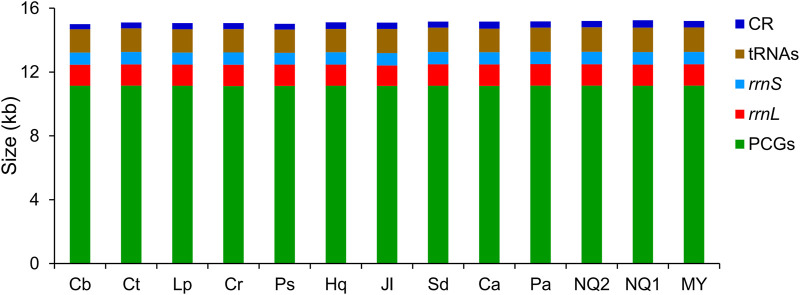
Size comparison of protein-coding genes (PCGs), transfer RNA genes (tRNAs), *rrnL*, *rrnS*, and control region (CR) among 13 Lycaenidae mitogenomes. Species are abbreviated as follows: Cb, *Curetis bulis*; Ct, *Cigaritis takanonis*; Lp, *Lycaena phlaeas*; Cr, *Coreana raphaelis*; Ps, *Protantigius superans*; Hq, *Hypaurotis quercus*; Jl, *Japonica lutea*; Sd, *Shijimiaeoides divina*; Ca, *Cupido argiades*; Pa, *Polyommatus amorata*; NQ2, *Agriades orbitulus* NQ2; NQ1, *Agriades orbitulus* NQ1; MY, *Agriades orbitulus* MY.

In Lycaenidae, 13 PCGs began with an ATN codon, except for *cox1* (which started with CGA), and terminated with complete (TAA or TAG) or truncated (TA or T) stop codons. Twenty-three genes (nine PCGs and 14 tRNAs) were located on the J-strand, with the remaining 14 genes encoded on the N-strand. The conservation in sequence size was observed in CRs, tRNAs, *rrnS*, *rrnL*, and PCGs among lycaenid mitogenomes, with the minimum variation observed in PCGs (27 bp) and the maximum in CRs (139 bp) (Figure 1).

**FIGURE 2:**
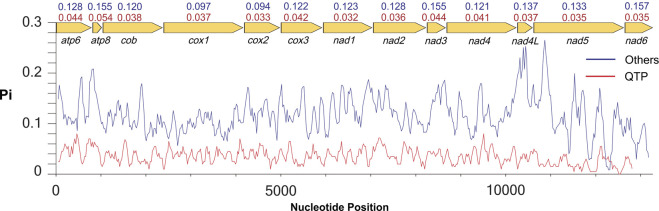
Nucleotide diversity (Pi) of 13 PCGs in 13 sequences of Lycaenidae. Lines show the values of Pi in a sliding window analysis. The red line represents the QTP species, and the blue line represents other species. The Pi values for each group and gene are shown on the gene name. Red values represent the QTP species, and blue values represent other species.

Nucleotide diversity in 13 lycaenid PCGs differed considerably among taxa and among genes. Nucleotide diversity estimates were significantly lower in QTP species than in other taxa, with *atp8* (*π* = 0.054) exhibiting the highest polymorphism and *nad1* (*π* = 0.032) showing the lowest (Figure 2). In other species, diversity was highest and lowest in *nad6* (*π* = 0.157) and *cox2* (*π* = 0.094), respectively (Figure 2).

### 3.2 Nucleotide composition and codon usage

**FIGURE 3:**
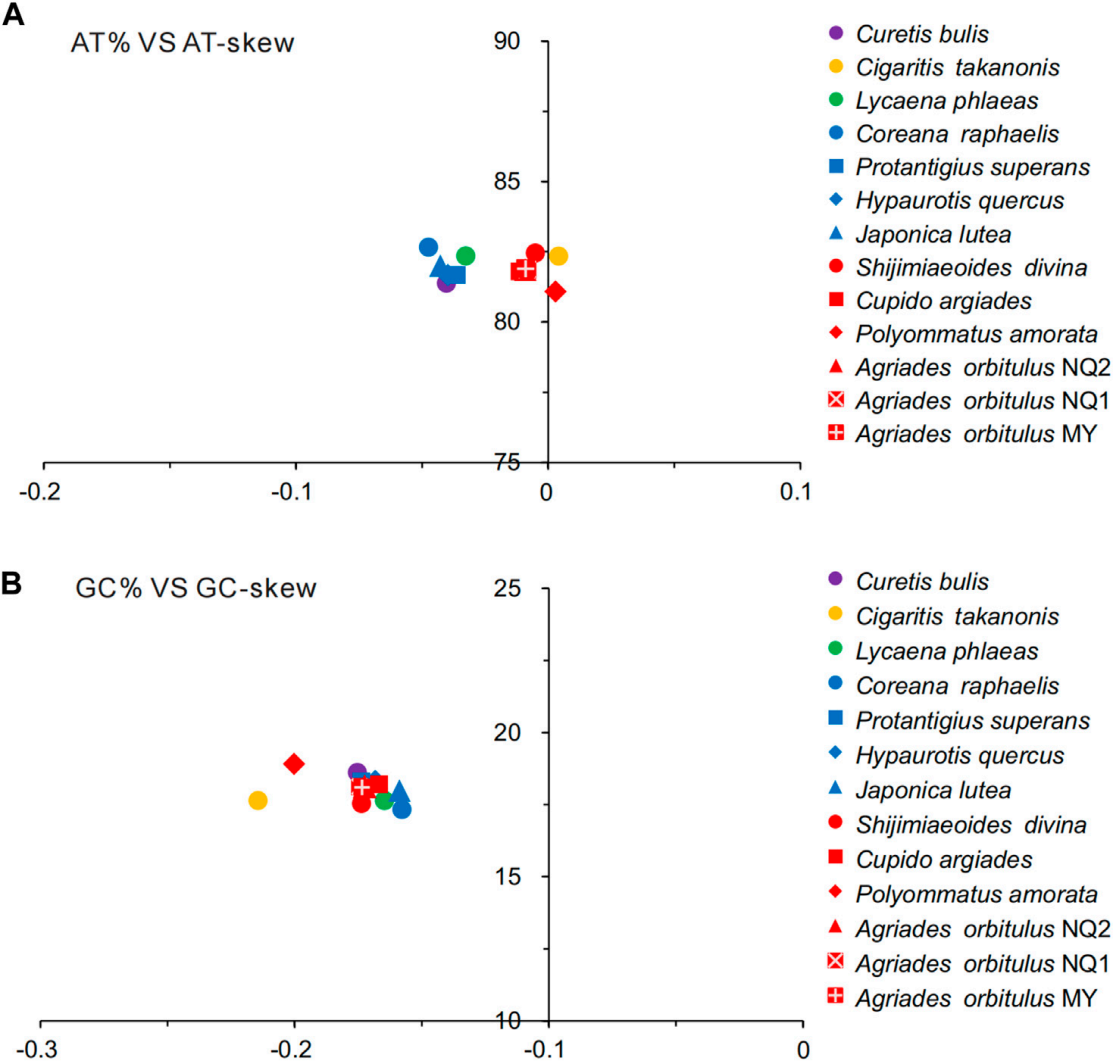
AT% vs. AT-skew **(A)** and GC% vs. GC-skew **(B)** in the 13 Lycaenidae mitochondrial genomes. Results are shown as the bp percentage (*Y*-axis) and nucleotide skews (*X*-axis). Values are calculated for J-strands for full-length mitogenomes.

**FIGURE 4:**
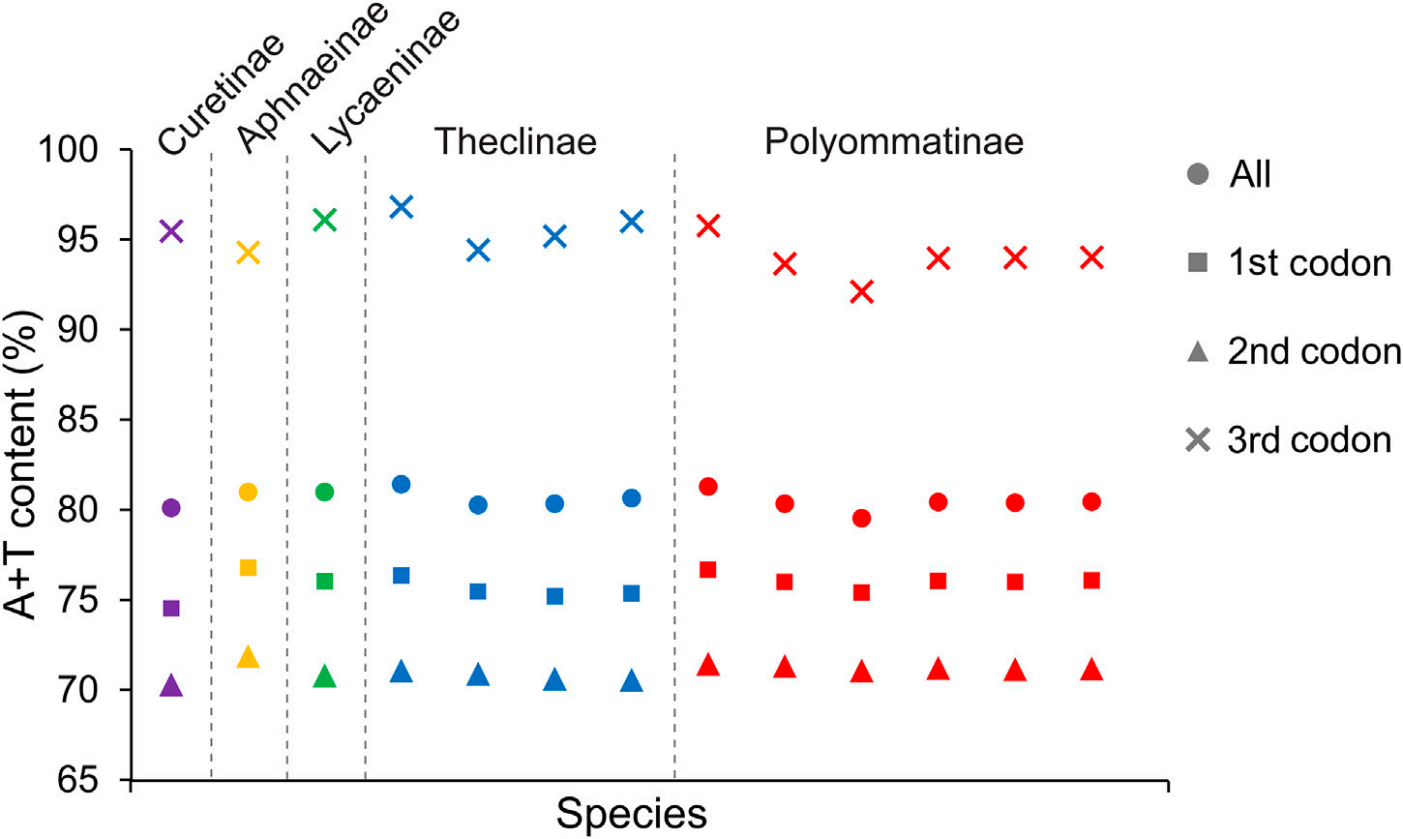
A + T contents of mitochondrial protein-coding genes among five subfamilies within Lycaenidae.

All Lycaenidae mitogenomes on the J-strand presented a similar nucleotide composition, characterized by a high A + T content, moderate negative AT skews (−0.0475 to 0.0041), and consistently negative GC skews (−0.1578 to −0.2145). Summaries of A + T% vs. AT-skew and G + C% vs. GC-skew across all available complete mitogenomes of Lycaenidae are shown in Figure 3. Among the 13 mitochondrial sequences, the A + T content ranged from 81.08% (*P. amorata*) to 82.66% (*Coreana raphaelis*) and 11 sequences had negative AT-skews, while the other two were positive ( *Cigaritis takanonis* and *P. amorata*) (Figure 3). Moreover, a comparative analysis of Lycaenidae mitogenomes indicated that the A + T content was always highest in the CR (90.02%–94.61%) and lowest in PCGs (79.58%–81.46%). Furthermore, it differed substantially among codon positions of PCGs; in particular, the third codon position had a higher A + T content (92.17%–96.87%) than those of the first (74.58%–76.82%) and second (70.35%–71.92%) positions (Figure 4).

There was a small difference in the number of codons but a bias in codon usage (Supplementary Table S5). The number of codons in the 13 sequences ranged from 3,708 (*C. raphaelis*) to 3,717 (*A. orbitulus* MY and *A. orbitulus* NQ2). A relative synonymous codon use (RSCU) analysis showed that the mitogenomes used up to 59 invertebrate mitochondrial codons (in four sequences) and at least 51 codons (*C. raphaelis*) (Supplementary Table S5). In Lycaenidae, at least one of the five GC-rich codons CCG (P), GCG (A), CGC (R), CGG (R), and GGC (G) was not used, and the five AT-rich codons UUA (L), UUU (F), AUU (I), UAU (Y), and AUA (M) were the most frequently used codons (Supplementary Table S5).

**FIGURE 5:**
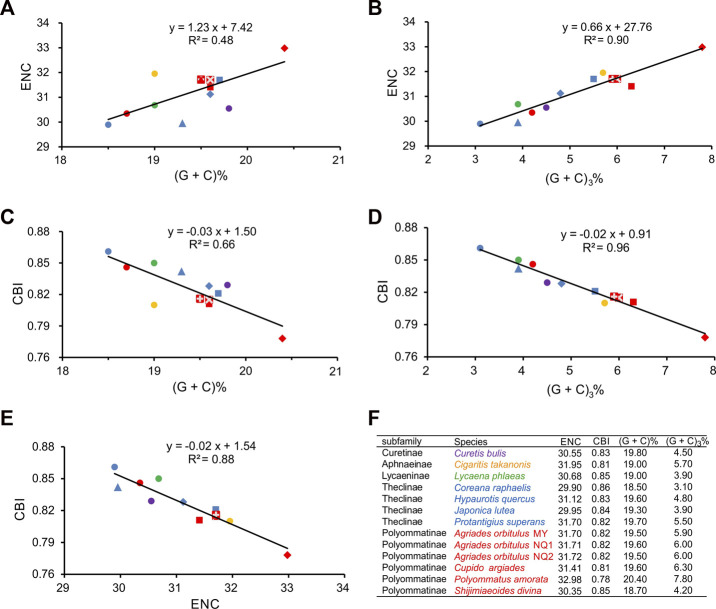
Evaluation of codon bias in the 13 Lycaenidae mitogenomes. (G + C)%, G + C content at all codon positions in the 13 protein-coding genes (PCGs). (G + C)_3_%, G + C content at the third codon positions in 13 PCGs. ENC, effective number of codons. CBI, codon bias index. The colors of symbols match those in Figure 3.

**FIGURE 6:**
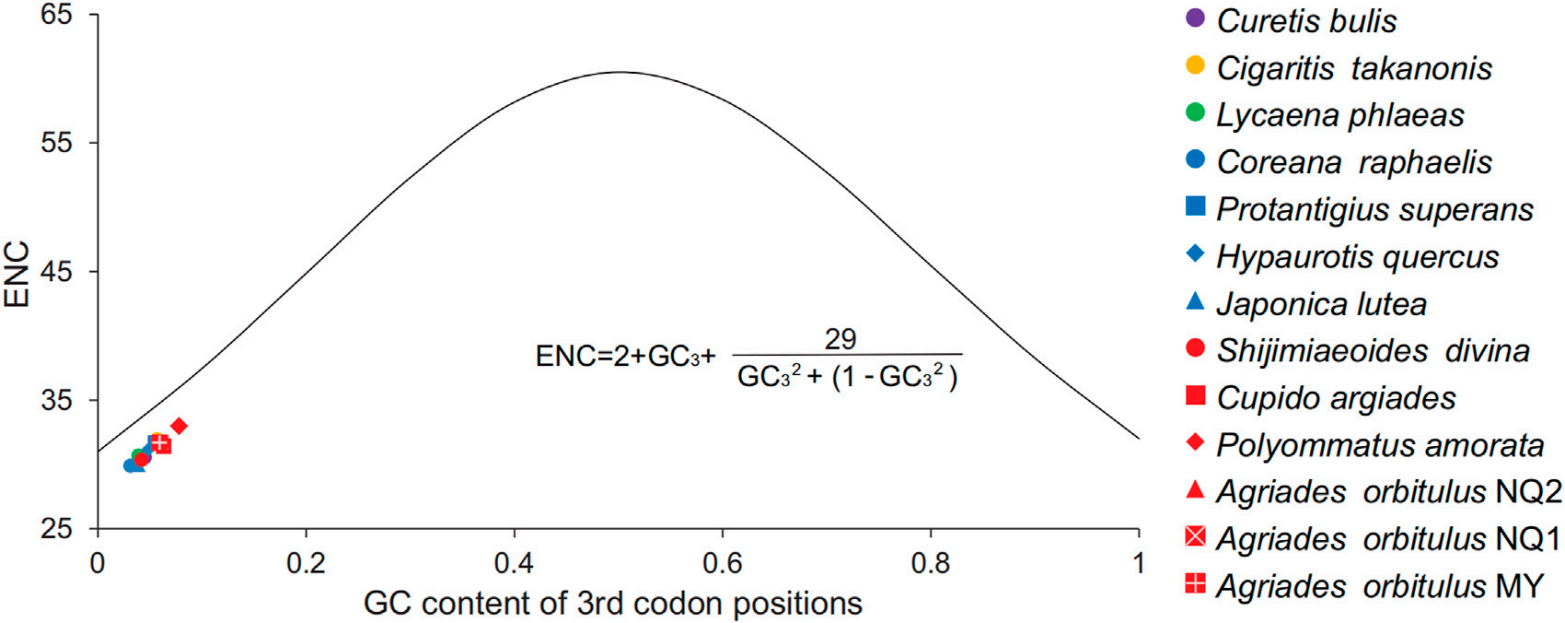
Correlation between the effective number of codons (ENC) and G + C content at the third codon position (GC_3_) in the 13 protein-coding genes (PCGs) for Lycaenidae species. The solid line represents the relationship between the ENC and GC_3_ content.

To further investigate codon usage bias among Lycaenidae species, we analyzed the correlation between the ENC, CBI, GC_a_, and GC_3_ for the 13 PCGs (Figure 5). The ENC ranged from 29.90 (*C. raphaelis*) to 32.98 (*P. amorata*), with an average of 31.21, and the CBI ranged from 0.78 (*P. amorata*) to 0.86 (*C. raphaelis*), with an average of 0.82. The ENC was positively correlated with GC_a_ (*R* ^2^ = 0.48, *p* < 0.01) (Figure 5A) and GC_3_ (*R* ^2^ = 0.90, *p* < 0.01) (Figure 5B). CBI was negatively correlated with GC_a_ (*R* ^2^ = 0.66, *p* < 0.01) (Figure 5C) and GC_3_ (*R* ^2^ = 0.96, *p* < 0.01) (Figure 5D), and the ENC and CBI were significantly negatively correlated (*R* ^2^ = 0.88, *p* < 0.01) (Figure 5E). In addition, the ENC and GC_3_ values were compared with those of the standard curve (ENC^*^, codon bias only determined by the base composition at the third position), and the actual sample points for all Lycaenidae fell far below the ENC^*^ (Figure 6).

### 3.3 Transfer RNA genes

**FIGURE 7:**
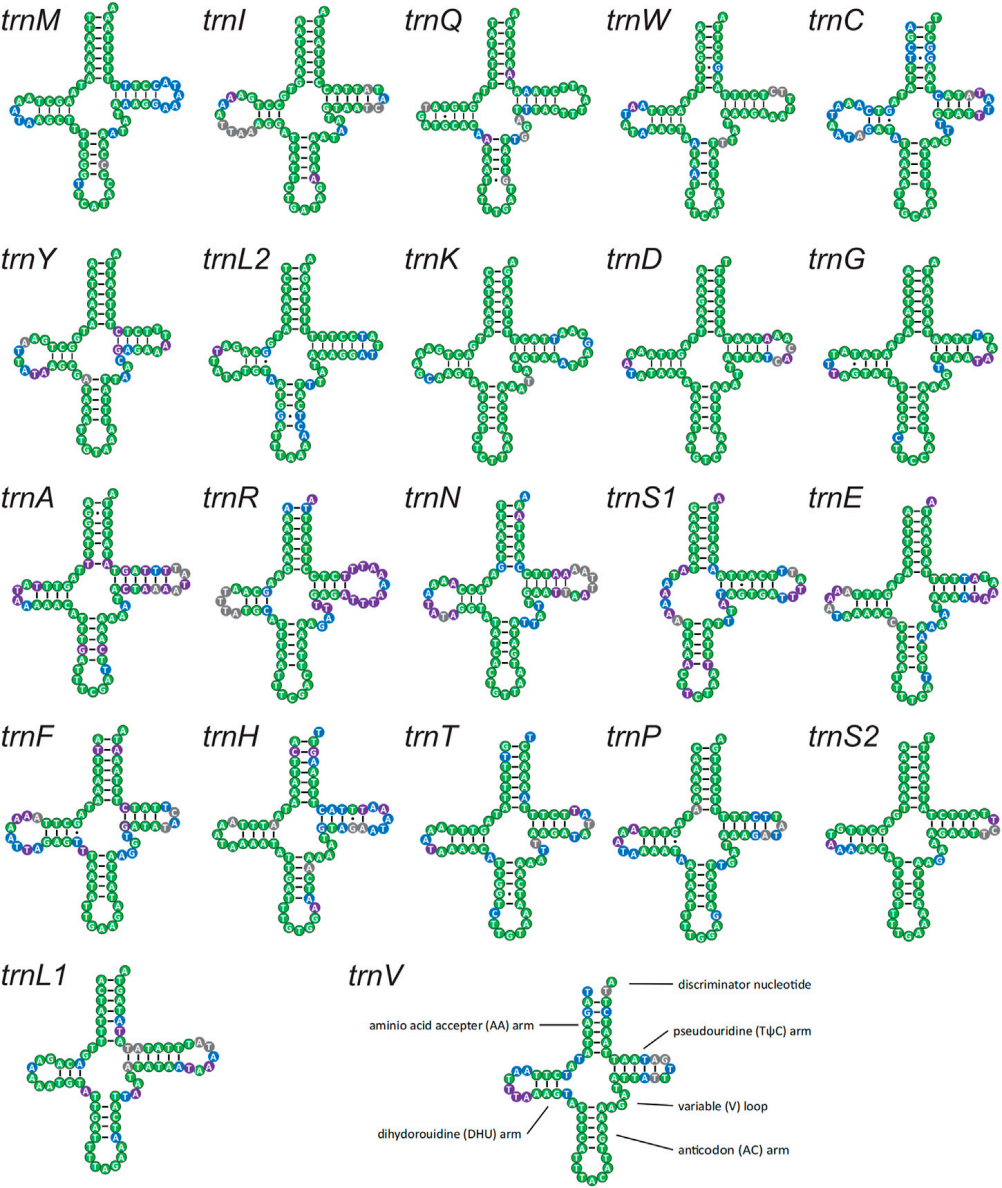
Putative secondary structures of the 22 tRNA genes identified in the mitochondrial genome of Lycaenidae. All tRNA genes are shown in the order of occurrence in the mitochondrial genome starting from *trnM*. The nucleotides showing 100% identity within Lycaenidae, Polyommatinae, and QTP species are marked in green, blue, and purple, respectively. Bars indicate Watson–Crick base pairings, and dots between G and T pairs mark canonical base pairings in tRNA.

Twenty-two tRNAs were detected in these four new mitogenomes and were conserved in sequence and structure in lycaenids (Figure 7). High conservation was confirmed at the family level (64.06%–92.86%), subfamily level (71.43%–98.57%), and QTP species level (89.06%–100.00%). A stable canonical cloverleaf secondary structure was forecasted. Variations in *trnS1* were detected in the absence of the dihydrouridine arm, the type of anticodon, and copy number, and this variation may be associated with environmental adaptation.

The anticodon of *trnS1* was not unique and varied from the most common TCT (10 sequences) to ACT (*Japonica lutea* and *Cupido argiades*) or GCT (*Curetis bulis*). In addition, reports of a second copy of *trnS1* with the anticodon ACT in two species (*C. raphaelis* and *Hypaurotis quercus*) suggested that multiple functional copies contribute to environmental adaptation.

### 3.4 Non-coding regions

The largest non-coding region of Lycaenidae with a length of 324 bp (*Curetis bulis*) to 463 bp (*A. orbitulus* NQ1) was the CR, located at a conserved position between *rrnS* and *trnM*. The CR had special structures that make it the starting point of DNA replication, and the following three characteristics were observed in 13 Lycaenidae sequences. 1) Each sequence had at least one maximal tandem repeat unit ranging from 12 to 24 bp in length, with a maximum length of 24 bp in *C. raphaelis*. Only two perfect repeats were observed for each tandem repeat unit in 13 sequences, with imperfect repeats in few species (Supplementary Table S6). 2) The longest TATA motif of 56 bp was observed in *C. raphaelis*, and the motif was lacking in *Lycaena phlaeas*. 3) A large poly(T/A) motif with a length of 19 or 20 bp and a small (T/A) motif with a length of 7–10 bp were observed in all sequences.

**FIGURE 8:**
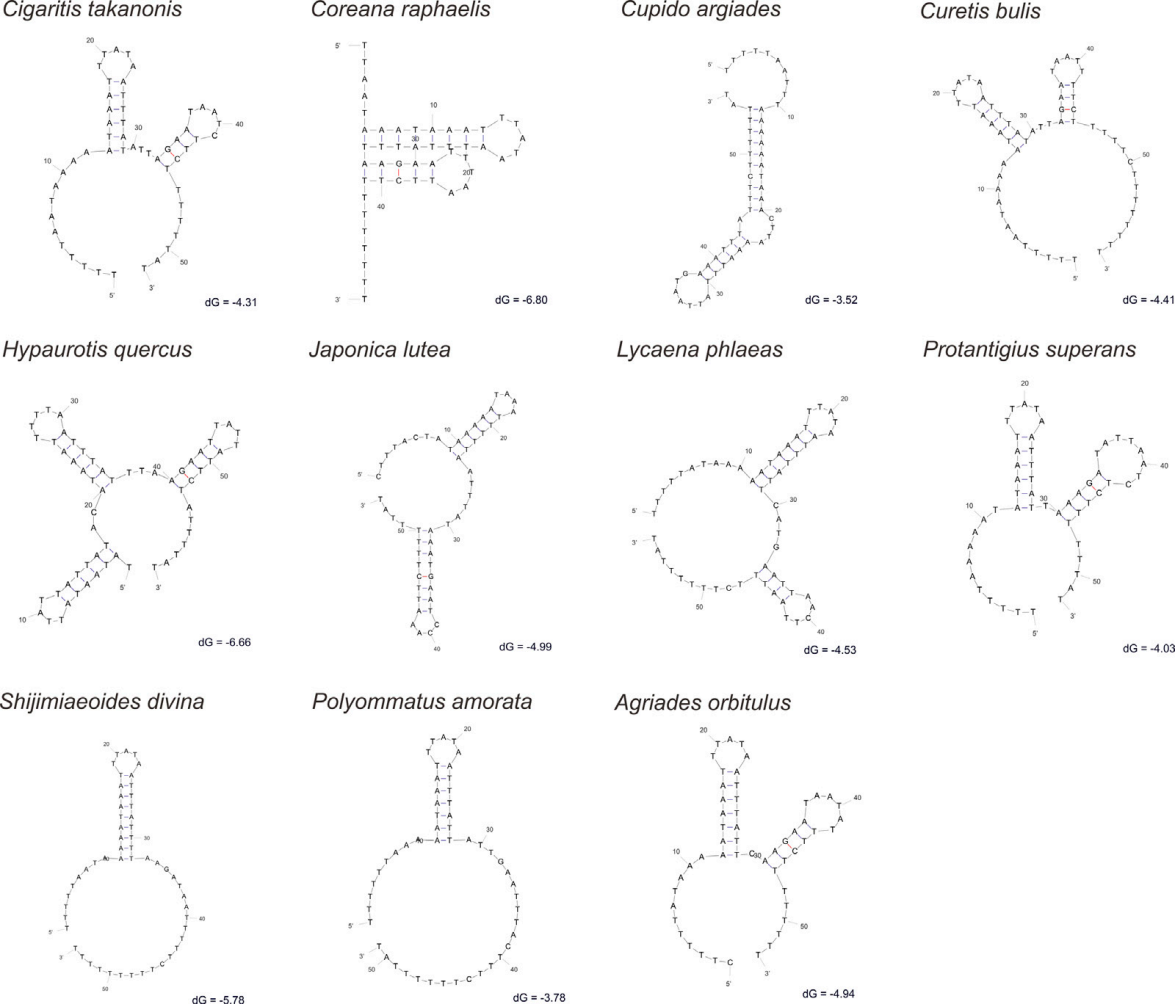
Non-coding region between *trnQ* and *nad2* in Lycaenidae mitogenomes. dG, free energy, kcal/mol. The most stable structure was selected.

**FIGURE 9:**
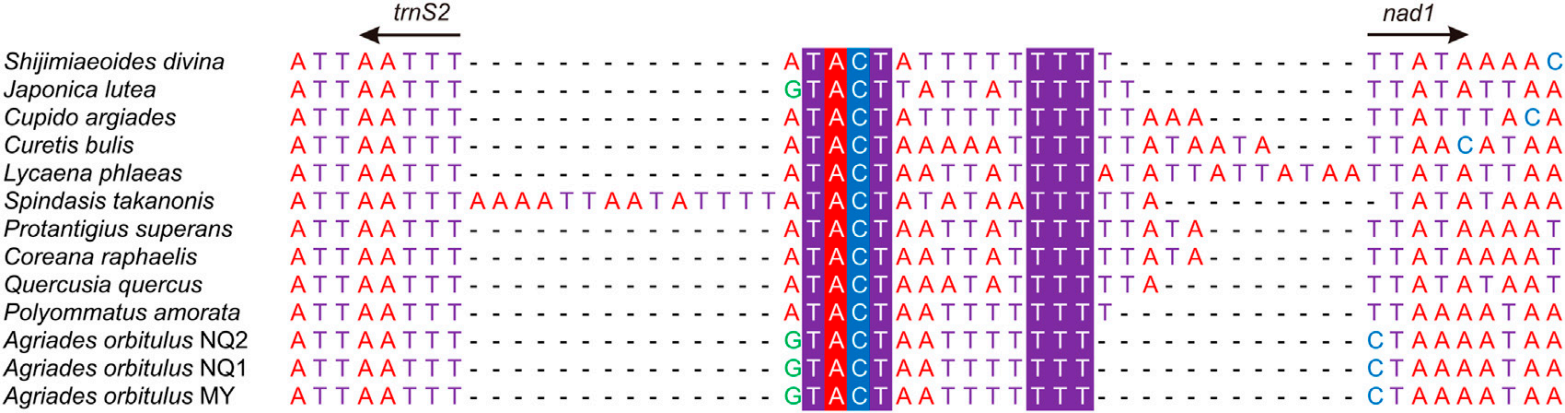
Non-coding region between *trnS2* and *nad1* in the 13 Lycaenidae mitogenomes. Conserved bases in the 13 Lycaenidae are highlighted.

Additionally, many IGRs were detected in Lycaenidae, besides the CR. Thirteen sequences shared two IGRs (*trnQ-nad2* and *trnS2-nad1*) (Figures 8, 9). Special structural domains were found in three IGRs (*trnE-trnF*, *trnS1-trnE*, and *trnP-nad6*) in two QTP species (Supplementary Figure S1), and two relatively large IGRs (*nad6-cob* and *cob-trnS2*) were found in two QTP species.

Seven IGRs could be characterized as follows. 1) The secondary structures of *trnQ-nad2* (48–60 bp) were predicted for 13 sequences, revealing the typical stem-loop structure (Figure 8). 2) Two conserved motifs were observed at ends, “TACT” near *trnS2* and “TTT” near *nad1*, the predicted binding site of the *Drosophila* mitochondrial transcription termination factor (DmTTF). The middle parts of the two motifs were composed of A and T in 13 lycaenids at *trnS2-nad1* (15–31 bp) (Figure 9). 3) A 27-bp motif of (TAT)2, (TA)8, and “CATAT” was observed in two QTP species but did not exceed 3 bp in non-QTP species at *trnE*-*trnF* (29–39 bp) (Supplementary Figure S1A). 4) A 10-bp motif of “TTATATTATT” was observed in QTP species and was not detected in non-QTP species (no more than 11 bp) at *trnS1*-*trnE* (12–30 bp) (Supplementary Figure S1B). 5) A 7-bp sequence of “ATTTGAT” was observed in two QTP species, while other species had only 2 bp at *trnP*-*nad6* (Supplementary Figure S1C). 6) A 6-bp sequence was observed in QTP species but was lacking in non-QTP species at *nad6-cob*. 7) A short sequence (2–10 bp) was observed at *cob-trnS2* only in QTP species.

Furthermore, a large IGR was detected in *A. orbitulus* at *cox3*-*trnG* (29 bp); this may be a species-specific sequence and was likely to have a stem-loop structure (Supplementary Figure S2). Other non-coding regions showed substantial variation in size and shared similar characteristics, including a high A + T content and poly(T/A), AT motif, or stem-loop structures.

### 3.5 Mitochondrial phylogeny

**FIGURE 10:**
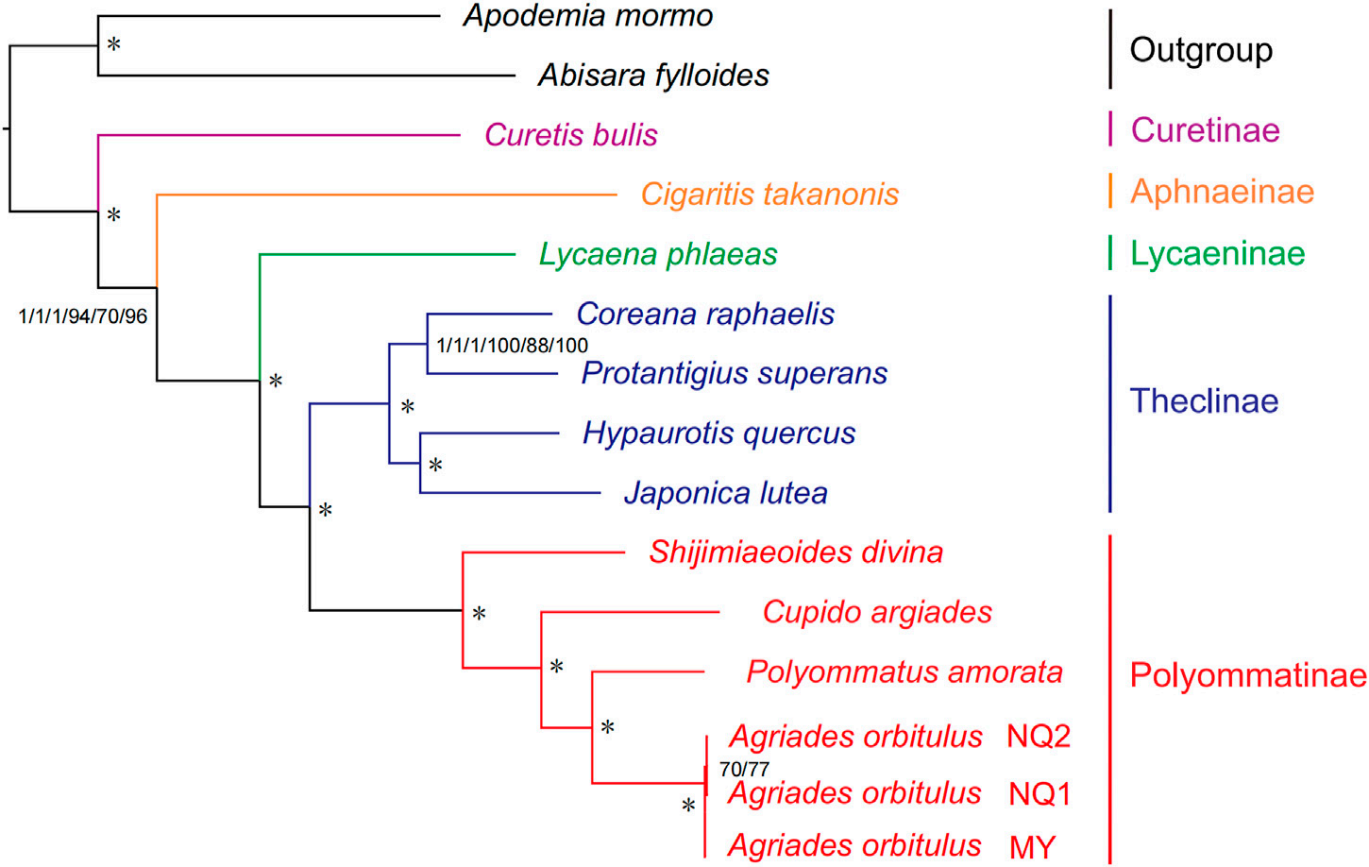
Phylogenetic relationships among five subfamilies within Lycaenidae inferred from mitogenomic data. Numbers from left to right are Bayesian posterior probabilities (PP) and ML bootstrap (BS) values for each of the three datasets (P123, P123AA, and P123RNA). Asterisk (*) indicates PP = 1.0 and BS > 90. Full phylogenetic results are provided in Supplementary Figure S3.

BI and ML methods for phylogenetic reconstruction based on mitogenomes yielded similar tree topologies for Lycaenidae at the species level (Figure 10; Supplementary Figure S3). The only incongruence was the relationship among *A. orbitulus* NQ1, *A. orbitulus* NQ2, and *A. orbitulus* MY (Supplementary Figure S3). Five subfamilies (Curetinae, Aphnaeinae, Lycaeninae, Theclinae, and Polyommatinae) in Lycaenidae were recovered consistently with high support (Figure 10; Supplementary Figure S3). These clades were identified as monophyletic groups. Curetinae was placed at the basal position in Lycaenidae, followed by the divergence of Aphnaeinae and Lycaeninae, and Theclinae clustered with Polyommatinae (Figure 10). Furthermore, the relationship among the genera in Polyommatinae were well-supported by both BI and ML analyses, indicating the basal position of *Shijimiaeoides* and the following topology: *Shijimiaeoides divina* + (*Cupido argiades* + (*P. amorata* + *A. orbitulus*)) (Figure 10).

### 3.6 Adaptive evolution of protein-coding genes

**FIGURE 11:**
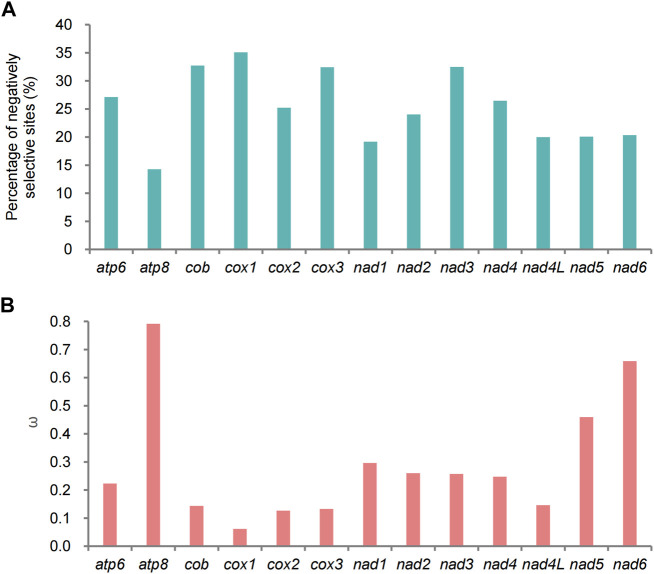
Results of selective pressure analyses. **(A)** Percentage of sites under negative selection in each of the 13 protein-coding genes (PCGs) in Polyommatinae determined by FEL site-by-site analyses. **(B)** Ratios (*ω*) of non-synonymous substitutions to synonymous substitutions for each of the 13 PCGs in Polyommatinae.

The *ω* values of 13 PCGs were low (*ω* < 1), suggesting that these genes were under purifying selection (Figure 11B). The lowest *ω* value was presented in *cox1,* and *atp8* showed the highest *ω* value (Figure 11B). This was also true for the percentage of negatively selective sites (Figure 11A). In the branch-specific model, only two PCGs (*atp8* and *nad6*) were significantly different between the QTP lycaenid species and non-QTP species (Supplementary Table S7). In the branch-site model analysis, signatures of positive selection were detected at one codon (position 404) of *cox1*, approaching significance (*p* = 0.055) (Supplementary Table S8), suggesting that this locus may be associated with high-altitude adaptation. The selected codon changed from CCT (Pro) to AAT (Asn) in the QTP species. We detected no evidence of positive selection in other positions or genes; however, the role of positive selection cannot be excluded.

## 4 Discussion

### 4.1 General features of Lycaenidae mitogenomes are conserved

The mitogenomes of animals are generally conserved, reflecting their indispensable functions. However, adaptive evolution may alter mitogenomic characteristics (Li et al., 2016; Yuan et al., 2020), resulting in variation in the size, number, and arrangement of genes (Adams and Palmer, 2003; Wang et al., 2014a; Gan et al., 2016; Chen et al., 2020c). In Lycaenidae, mitogenomes are highly conserved in size, structure, and function (Kim et al., 2011; Zhang et al., 2013; Jeong et al., 2017), as supported by analyses of gene content, gene arrangement, base composition, codon usage, and transfer RNAs. No rearrangements were detected (Boore, 1999).

The nucleotide composition affects codon usage, and different codon usage patterns may lead to differences in biological functions (Foroughmand-Araabi et al., 2015; Ma et al., 2015; Zhou et al., 2021). In Lycaenidae, a strong bias in nucleotide composition (toward A and T) was found, and this pattern is common in insects (Meng et al., 2022; Qi et al., 2022; Sun et al., 2022). Moreover, this codon bias can be explained by broad patterns in both natural selection and mutation.

Transfer RNA genes were highly conserved and exhibited a typical secondary cloverleaf structure. *TrnS1* exhibited variation in the presence of the dihydrouridine arm, type of anticodon, and copy number, considered an important indicator of adaptive evolution. In insects, the lack of the dihydrouridine arm in tRNA is not rare (Yang et al., 2019c; Wei et al., 2021; Wu et al., 2022), and isoacceptor tRNAs (which have a different anticodon but charge the same amino acid) may be related to codon usage patterns and selection (Oliveira et al., 2008; Behura et al., 2010). Moreover, observations of more than 22 tRNAs had been reported in only a few insect mitogenomes. In Lycaenidae, two *trnS1* genes, one copy containing the anticodon TCT and another copy containing the anticodon ACT, have been observed in *C. raphaelis* and *H. quercus* (Kim et al., 2006). Therefore, the observation of multiple copies of *trnS1* in *P. amorata* was reasonable, and the second copy may be functional (Behura et al., 2010; Behura and Severson, 2011; Pang et al., 2014; Zhou et al., 2020). The anticodon of the second copy of *trnS1* was ACT in *P. amorata* and was expected to have a clover structure without the DHU arm. The second copy showed 77.78% sequence identity to the first copy (determined using BLAST as the ratio of matched bases to the total number of aligned bases) (Supplementary Figure S4). These features were important indicators of adaptive evolution in Lycaenidae tRNA, though we cannot exclude the role of random ancestral mutations. Further research including additional lycaenid species and individuals are needed to clarify the strength and role of adaptive evolution.

### 4.2 Mitogenomic data can provide valid phylogenetic signals for Lycaenidae

Mitogenomes are relatively stable and have excellent characteristics for evolutionary analyses (Yuan et al., 2015; Li et al., 2019; Li et al., 2022), as demonstrated in many lepidopteran groups (Liu et al., 2016; Shi et al., 2020; Sun et al., 2020). Subfamilies in Lycaenidae formed monophyletic groups in the phylogeny. The classification of some species in the family is controversial, mainly stemming from disagreements in analyses based on morphological characteristics (Ugelvig et al., 2011). Despite variation in phylogenetic relationships inferred from morphology, analyses based on molecular data yield highly consistent results. Therefore, scholars have suggested using a combination of morphological and molecular data to confirm the relationships among groups in Lycaenidae (Wahlberg et al., 2005; Stekolnikov et al., 2013; Talavera et al., 2013). A subfamily-level phylogeny of Lycaenidae is clearly defined as [Curetinae + (Aphnaeinae + (Lycaeninae + (Theclinae + Polyommatinae)))], providing an updated view of relationships within this family. The phylogenetic analysis of Lycaenidae is consistent with previous research supported by molecular data, showing that mitogenomes provide useful data for resolving phylogenetic relationships (Jeong et al., 2017; Zhou et al., 2020). Considering the limited taxon sampling in the present study, additional mitogenomes covering more subfamilies/tribes (especially Theclinae and Polyommatinae) will be necessary to improve our understanding of mitogenomic phylogeny in Lycaenidae.

### 4.3 Adaptive evolution of Lycaenidae mitogenomes

Generally, mitochondrial genes show variation in evolutionary rates, reflecting differences in selective pressure (Chen et al., 2018; Sarvani et al., 2018; Sarkar et al., 2020). Low *d*_N_/*d*_S_ (*ω* < 1) ratios for 13 PCGs implied that the mitogenomes underwent purifying or stabilizing selection. Actually, a harsh high-altitude environment would shrink the effective population size, resulting in decreased selection on mutations but increased effects of genetic drift (Gomaa et al., 2011). Therefore, the net effect of genetic drift can lose genetic diversity. In this case, the reduced *d*_N_/*d*_S_ ratios might not be directly associated with high-altitude adaptation.

Additionally, it is generally believed that the functional importance of a gene dictates its level of conservation. This can explain the high conservation of *cox1*, a subunit of cytochrome oxidase, which is the fourth central enzyme complex of the respiratory electron transport chain and plays a crucial role in metabolism. The evolutionary rate of *cox1* was the slowest among genes in this study, suggesting that purifying selection played a crucial role in its evolution, consistent with results for other insect species (Singh et al., 2017; Chen et al., 2020b; Yuan et al., 2020; Liu et al., 2022b). The harsh environment of the QTP may lead to strong selection pressure. In particular, mutations in genes related to oxygen use may be favored in the QTP. We detected a relatively significant signature of selection (*p* = 0.0550) in *cox1* and found that non-synonymous substitutions affected the protein structure. The amino acid at residue 404 changed from proline (Pro, P) to asparagine (Asn, N), classified as a missense mutation. Missense mutations can cause the polypeptide chain to lose its original function and lead to protein abnormalities (Henderson et al., 2010; Beer et al., 2013; Caporali et al., 2018). Nevertheless, these were identified as beneficial mutations in this study and may be the key to high-altitude adaptation. The evolutionary process and the role of this beneficial missense mutation need to be verified by experiments.

Moreover, *cox1* is used as a DNA barcode for species identification (Hebert et al., 2003). DNA barcoding is widely used in taxonomies (Bakhoum et al., 2018; Choudhary et al., 2018; DeSalle and Goldstein, 2019; Bianchi and Goncalves, 2021) and has been applied to many groups in Lepidoptera (Janzen et al., 2005; Song et al., 2014; Efetov et al., 2019). Therefore, the high conservation of *cox1* in our study suggests that it can also be used for DNA barcoding in Lycaenidae. However, the accuracy of *cox1* for species identification in Lycaenidae still needs more evaluation using experimental data, and its utility in phylogeny, population genetics, and phylogeography needs to be further verified.

In addition to PCGs, our research suggested that non-coding regions also underwent positive selection in the high-altitude environment. The largest non-coding region was the CR in mitogenomes, considered the start of transcription and showed typical structural characteristics similar to those in other animal mitogenomes (Yu et al., 2015; Sun et al., 2016; Yang et al., 2019b). IGR in *trnQ*-*nad2* has been observed not only in Lycaenidae but also in most Lepidopteran species (Kim and Kim, 2016; Laemmermann et al., 2016; Kim et al., 2017; Sivasankaran et al., 2017; Wang et al., 2022b), and the stem-loop structure may also be widespread in Lepidoptera. IGR in *trnS2-nad1* was observed in Lycaenidae, considered DmTTF, found in other insects (Beckenbach, 2012; Wang et al., 2014b). Intergenic motifs significantly longer than those in other species were found in some species of Lycaenidae, and these may be specific fragments, e.g., a 64-bp sequence in *trnK-trnD* of *L. phlaeas* (Zhang et al., 2013), a 26-bp sequence in *trnR-trnN* of *Curetis bulis* (Zhang et al., 2013), and a 29-bp sequence in *cox3-trnG* of *A. orbitulus*. These findings provide evidence for positive selection in non-coding regions.

Non-coding regions may also experience selection in the high-altitude QTP, as evidenced by the extension of non-coding fragments in QTP species and emergence of special structures. Analyses of all particular IGRs provide insight into evolutionary processes, which can be divided into four stages. In the first stage, the non-coding region and *nad6-cob* and *cob-trnS2* IGRs arose. IGRs of 2–10 bp size arose in QTP species, while no or several nucleotides overlapped in other Lycaenidae. In the second stage, non-coding regions were extended. The size of *trnP-nad6* IGR increased from 2 bp to 7 bp. In the third stage, secondary structures began to appear; however, the structures were not stable. Small units of “TA” and “TTA” and possible structural features appeared in QTP species at the *cox3-trnG* IGR, and a 10-bp motif and possible structure appeared in QTP species at the *trnS1-trnE* IGR. In the final stage, stable structures appeared. Between *trnE* and *trnF*, the secondary structure of the sequence could be predicted. The large non-coding region in *trnE-trnF* was considered DmTTF and had a similar structure to that of other insect species (Roberti et al., 2003; Beckenbach, 2012; Yuan et al., 2016; Wang et al., 2022a). Other insect groups also had the *trnE-trnF* IGR and other structural features consistent with repeat regions (Wu et al., 2012; Zhang et al., 2014; Chen et al., 2020a).

Research on IGRs has focused on large fragments (CR, *trnE-trnF* IGR, and *trnS2-nad1* IGR) and taxa-specific fragments, with relatively little research on the evolution of small or medium-sized IGRs. These IGRs could provide important evidence for understanding the contribution of non-coding regions to adaptive evolution. The results of this study provide new insight into the adaptability of mitogenomes in extreme environments, like the QTP.

Mitochondrial hypoxia-related adaptive evolution can result in metabolic alterations that promote survival, to some extent. These adaptive changes, e.g., changes in the structure and function of proteins associated with oxidative phosphorylation (Yang et al., 2019a; Bartakova et al., 2021), physiological variation (Scott et al., 2018; Facultative myrmecophily (hymenopteraDawson and Scott, 2022), and metabolite changes (Bacchiocchi and Principato, 2000), are regulated by hypoxia-inducible factor and gene expression (Taylor, 2008; Goda and Kanai, 2012; Scott et al., 2018). The underlying mechanisms differ between groups (Mahalingam et al., 2017). PCGs and non-coding regions involved in mitogenome evolution in high-altitude conditions in Lycaenidae exhibit these changes in protein structure and function and are directly regulated by DmTTF. The shared characteristics in the two QTP species could be associated with high-altitude adaptation; however, elaborating on how the role of ancestral polymorphisms needs to be tested in future studies by using intensive taxon sampling and population genetic analysis.

## 5 Conclusion

In this study, we sequenced four mitogenomes of two Lycaenidae species inhabiting the QTP. The gene content, gene arrangement, base composition, codon usage, and transfer RNA genes in the sequence and structure showed high conservation within Lycaenidae. Mitogenomic data could provide effective phylogenetic signals for Lycaenidae. Both PCGs and non-coding regions were associated with high-altitude adaptation. Future studies including more mitogenomes and functional analyses of genes under positive selection and non-coding regions associated with environmental adaptation will improve our understanding of Lycaenidae evolution.

## Data Availability

The datasets presented in this study can be found in online repositories. The names of the repository/repositories and accession number(s) can be found below: https://www.ncbi.nlm.nih.gov/, ON411617-20.
