# Supplementary material for: Comparative mitogenomic and evolutionary analysis of Lycaenidae (Insecta: Lepidoptera): Potential association with high-altitude adaptation
Source: Front Genet. 2023 Apr 18;14:1137588. doi: 10.3389/fgene.2023.1137588 (PMC10151513; doi:10.3389/fgene.2023.1137588)
Supplement: Supplementary file 1 [file DataSheet1.ZIP › Supplemental Materials Revised/Figure S1 Three specific non-coding regions.pdf]

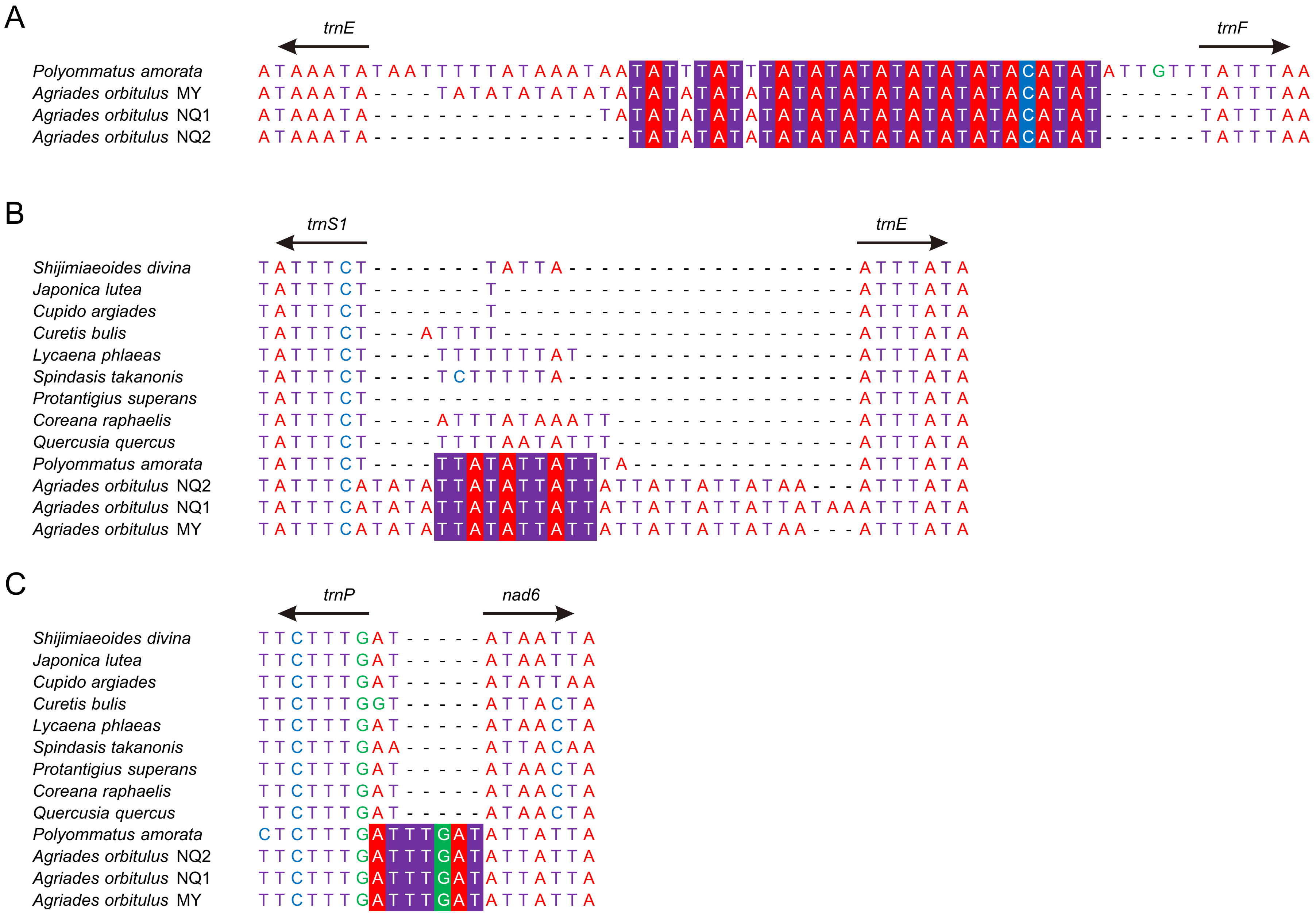
A
trnE
trnF
Polyommatus amorata ATAAATATAATTTTTATAAATAATATTTATTTATATATATATATATATACATATATTGTTTATTTAA
Agriades orbitulus MY ATAAATA----TATATATATATATATATATATATATATATATATATATATACATAT------TATTTAA
Agriades orbitulus NQ1 ATAAATA---------------TATATATATATATATATATATATATATATACATAT------TATTTAA
Agriades orbitulus NQ2 ATAAATA-----------------TATATATATATATATATATATATATATACATAT------TATTTAA
B
trnS1
trnE
Shijimiaeoides divina TATTTCT--------TATTA-------------------ATTTATA
Japonica lutea TATTTCT--------T-----------------------ATTTATA
Cupido argiades TATTTCT--------T-----------------------ATTTATA
Curetis bulis TATTTCT---ATTTT------------------------ATTTATA
Lycaena phlaeas TATTTCT----TTTTTTTAT-------------------ATTTATA
Spindasis takanonis TATTTCT----TCTTTTTA--------------------ATTTATA
Protantigius superans TATTTCT--------------------------------ATTTATA
Coreana raphaelis TATTTCT----ATTTATAAATT-----------------ATTTATA
Quercusia quercus TATTTCT----TTTTAATATTT-----------------ATTTATA
Polyommatus amorata TATTTCT----TTATATTATTTA----------------ATTTATA
Agriades orbitulus NQ2 TATTTCATATATTATATTATTATTATTATTATAA---ATTTATA
Agriades orbitulus NQ1 TATTTCATATATTATATTATTATTATTATTATTATAAATTTATA
Agriades orbitulus MY TATTTCATATATTATATTATTATTATTATTATAA---ATTTATA
C
trnP
nad6
Shijimiaeoides divina TTCTTTGAT-----ATAATTA
Japonica lutea TTCTTTGAT-----ATAATTA
Cupido argiades TTCTTTGAT-----ATATTAA
Curetis bulis TTCTTTGGT-----ATTACTA
Lycaena phlaeas TTCTTTGAT-----ATAACTA
Spindasis takanonis TTCTTTGAA-----ATTACAA
Protantigius superans TTCTTTGAT-----ATAACTA
Coreana raphaelis TTCTTTGAT-----ATAACTA
Quercusia quercus TTCTTTGAT-----ATAACTA
Polyommatus amorata CTCTTTGATTTGATATTATTA
Agriades orbitulus NQ2 TTCTTTGATTTGATATTATTA
Agriades orbitulus NQ1 TTCTTTGATTTGATATTATTA
Agriades orbitulus MY TTCTTTGATTTGATATTATTA
