# Supplementary material for: Comparative mitogenomic and evolutionary analysis of Lycaenidae (Insecta: Lepidoptera): Potential association with high-altitude adaptation
Source: Front Genet. 2023 Apr 18;14:1137588. doi: 10.3389/fgene.2023.1137588 (PMC10151513; doi:10.3389/fgene.2023.1137588)
Supplement: Supplementary file 1 [file DataSheet1.ZIP › Supplemental Materials Revised/Table S1 Sample information.docx]

**Table S1** Details sampling information in this study.

| Species | Sampling locality | Specimen voucher | Coordinate | Altitude (m) |
| --- | --- | --- | --- | --- |
| *Polyommatus amorata* | Menyuan County, Qinghai Province, China | MY-Po | 37°22'12"N, 101°37'12"E | 2900 |
| *Agriades orbitulus* NQ2 | Naqu County, Tibet Autonomous Region, China | NQ-Ao | 31°28'12"N, 92°06'00"E | 4500 |
| *Agriades orbitulus* NQ1 | Naqu County, Tibet Autonomous Region, China | NQ-Cs | 31°28'12"N, 92°06'00"E | 4500 |
| *Agriades orbitulus* MY | Menyuan County, Qinghai Province, China | MY-Ao | 37°22'12"N, 101°37'12"E | 2900 |
