# Supplementary material for: Comparative mitogenomic and evolutionary analysis of Lycaenidae (Insecta: Lepidoptera): Potential association with high-altitude adaptation
Source: Front Genet. 2023 Apr 18;14:1137588. doi: 10.3389/fgene.2023.1137588 (PMC10151513; doi:10.3389/fgene.2023.1137588)
Supplement: Supplementary file 1 [file DataSheet1.ZIP › Supplemental Materials Revised/Table S2 Species information.docx]

**Table S2** Characteristics of 13 mitogenomes from five subfamilies of Lycaenidae used in this study. Mitogenomes newly sequenced in this study are highlighted with an asterisk (*).

| Family | Subfamily | Species | Size (bp) | GenBank accession number |
| --- | --- | --- | --- | --- |
| Lycaenidae | Curetinae | *Curetis bulis* | 15162 | NC_023088 |
| Lycaenidae | Aphnaeinae | *Cigaritis takanonis* | 15349 | NC_016018 |
| Lycaenidae | Lycaeninae | *Lycaena phlaeas* | 15280 | NC_023087 |
| Lycaenidae | Theclinae | *Coreana raphaelis* | 15314 | NC_007976 |
| Lycaenidae | Theclinae | *Protantigius superans* | 15248 | NC_016016 |
| Lycaenidae | Theclinae | *Hypaurotis quercus* | 15366 | KM592971 |
| Lycaenidae | Theclinae | *Japonica lutea* | 15225 | NC_026517 |
| Lycaenidae | Polyommatinae | *Shijimiaeoides divina* | 15259 | NC_029763 |
| Lycaenidae | Polyommatinae | *Cupido argiades* | 15330 | NC_020779 |
| Lycaenidae | Polyommatinae | *Polyommatus amorata** | 15389 | ON411620 |
| Lycaenidae | Polyommatinae | *Agriades orbitulus* NQ2* | 15340 | ON411617 |
| Lycaenidae | Polyommatinae | *Agriades orbitulus* NQ1* | 15344 | ON411618 |
| Lycaenidae | Polyommatinae | *Agriades orbitulus* MY* | 15345 | ON411619 |
