# Supplementary material for: Comparative mitogenomic and evolutionary analysis of Lycaenidae (Insecta: Lepidoptera): Potential association with high-altitude adaptation
Source: Front Genet. 2023 Apr 18;14:1137588. doi: 10.3389/fgene.2023.1137588 (PMC10151513; doi:10.3389/fgene.2023.1137588)
Supplement: Supplementary file 1 [file DataSheet1.ZIP › Supplemental Materials Revised/Table S3 Saturation test.docx]

**Table S3** Substitution saturation tests for each of 13 protein-coding genes (PCGs), each of the three positions of concatenated13 PCGs, and each of two ribosomal RNA genes by using DAMBE. Iss, index of substitution saturation; Iss.cS, the critical Iss value.

| Dataset | Iss | Iss.cS | *P* value |
| --- | --- | --- | --- |
| *atp6* | 0.2063 | 0.7403 | ＜0.0001 |
| *atp8* | 0.3946 | 0.6630 | ＜0.0001 |
| *cob* | 0.1782 | 0.7710 | ＜0.0001 |
| *cox1* | 0.1396 | 0.7859 | ＜0.0001 |
| *cox2* | 0.1555 | 0.7389 | ＜0.0001 |
| *cox3* | 0.1741 | 0.7493 | ＜0.0001 |
| *nad1* | 0.1946 | 0.7608 | ＜0.0001 |
| *nad2* | 0.2296 | 0.7654 | ＜0.0001 |
| *nad3* | 0.2457 | 0.6955 | ＜0.0001 |
| *nad4* | 0.1990 | 0.7800 | ＜0.0001 |
| *nad4L* | 0.2385 | 0.6837 | ＜0.0001 |
| *nad5* | 0.2086 | 0.7910 | ＜0.0001 |
| *nad6* | 0.2937 | 0.7220 | ＜0.0001 |
| 13P123 | 0.1894 | 0.8399 | ＜0.0001 |
| Position 1st | 0.1466 | 0.8204 | ＜0.0001 |
| Position 2nd | 0.0642 | 0.8204 | ＜0.0001 |
| Position 3rd | 0.4595 | 0.8204 | ＜0.0001 |
| *rrnS* | 0.1787 | 0.7477 | ＜0.0001 |
| *rrnL* | 0.1963 | 0.7787 | ＜0.0001 |
