# Supplementary material for: Comparative mitogenomic and evolutionary analysis of Lycaenidae (Insecta: Lepidoptera): Potential association with high-altitude adaptation
Source: Front Genet. 2023 Apr 18;14:1137588. doi: 10.3389/fgene.2023.1137588 (PMC10151513; doi:10.3389/fgene.2023.1137588)
Supplement: Supplementary file 1 [file DataSheet1.ZIP › Supplemental Materials Revised/Table S4 Model selection.docx]

**Table S4** The best partitioning schemes and substitution models selected by IQ-TREE for the three datasets.

| Dataset | Subset | Best-fit scheme | Model |
| --- | --- | --- | --- |
| P123 | P1 | *atp6*_pos1, *cob*_pos1, *cox1*_pos1, *cox2*_pos1, *cox3*_pos1, *nad3*_pos1 | GTR+F+I+G4 |
|  | P2 | *atp6*_pos2, *cob*_pos2, *cox1*_pos2, *cox2*_pos2, *cox3*_pos2, *nad1*_pos2, *nad2*_pos2, *nad3*_pos2, *nad4*_pos2, *nad5*_pos2, *nad6*_pos2 | TIM+F+I+G4 |
|  | P3 | *atp6*_pos3, *atp8*_pos3, *cob*_pos3, *cox1*_pos3, *cox2*_pos3, *cox3*_pos3, *nad2*_pos3, *nad3*_pos3, *nad6*_pos3 | TIM+F+G4 |
|  | P4 | *atp8*_pos1, *atp8*_pos2, *nad1*_pos1, *nad2*_pos1, *nad4*_pos1, *nad4L*_pos1, *nad4L*_pos2, *nad5*_pos1, *nad6*_pos1 | TVM+F+G4 |
|  | P5 | *nad1*_pos3, *nad4*_pos3, *nad4L*_pos3, *nad5*_pos3 | TPM2u+F+I+G4 |
| P123RNA | P1 | *atp6*_pos1, *cob*_pos1, *cox1*_pos1, *cox2*_pos1, *cox3*_pos1, *nad3*_pos1 | GTR+F+I+G4 |
|  | P2 | *atp6*_pos2, *cob*_pos2, *cox1*_pos2, *cox2*_pos2, *cox3*_pos2, *nad1*_pos2, *nad2*_pos2, *nad3*_pos2, *nad4*_pos2, *nad5*_pos2, *nad6*_pos2 | TIM+F+I+G4 |
|  | P3 | *atp6*_pos3, *atp8*_pos3, *cob*_pos3, *cox1*_pos3, *cox2*_pos3, *cox3*_pos3, *nad2*_pos3, *nad3*_pos3, *nad6*_pos3 | TIM+F+G4 |
|  | P4 | *atp8*_pos1, *atp8*_pos2, *nad1*_pos1, *nad2*_pos1, *nad4*_pos1, *nad4L*_pos1, *nad4L*_pos2, *nad5*_pos1, *nad6*_pos1, *rrnL*, *rrnS* | TVM+F+I+G4 |
|  | P5 | *nad1*_pos3, *nad4*_pos3, *nad4L*_pos3, *nad5*_pos3 | TPM2u+F+I+G4 |
| 13P123AA | P1 | *atp6*, *atp8*, *cox2*, *nad1*, *nad2*, *nad3*, *nad4*, *nad4L*, *nad5*, *nad6* | mtMet+F+I+G4 |
|  | P2 | *cob*, *cox1*, *cox3* | mtART+I+G4 |
