# Supplementary material for: Comparative mitogenomic and evolutionary analysis of Lycaenidae (Insecta: Lepidoptera): Potential association with high-altitude adaptation
Source: Front Genet. 2023 Apr 18;14:1137588. doi: 10.3389/fgene.2023.1137588 (PMC10151513; doi:10.3389/fgene.2023.1137588)
Supplement: Supplementary file 1 [file DataSheet1.ZIP › Supplemental Materials Revised/Table S6 Maximum tandem repeats of control region.docx]

**Table S6** Maximum tandem repeats in control regions of 13 Lycaenidae mitogenomes.

| Species | Repeat sequence | Length | Perfect repetition | Imperfect repetition |
| --- | --- | --- | --- | --- |
| *Curetis bulis* | TTTTTTTTATTTT | 13 | 2 | 1 |
| *Cigaritis takanonis* | AAATTATTTATATATA | 16 | 2 | 1 |
| *Lycaena phlaeas* | TTTTTTTTTTATAT | 14 | 2 | - |
| *Coreana raphaelis* | AAATATTTAATATATATATATATA | 24 | 2 | - |
| *Protantigius superans* | ATAATATTAAAT | 12 | 2 | 2 |
|  | TTAATTAAATAT | 12 | 2 | - |
| *Hypaurotis quercus* | TTTAATTTATTAA | 13 | 2 | - |
|  | AATTAATTTATTT | 13 | 2 | - |
|  | TAAAAATTAATTT | 13 | 2 | 1 |
| *Japonica lutea* | TTATATATATATATAT | 16 | 2 | - |
| *Shijimiaeoides divina* | TTAATAAATTTT | 12 | 2 | 1 |
|  | AAATTTAATAAT | 12 | 2 | - |
| *Cupido argiades* | TATATATATATATATATA | 18 | 2 | - |
| *Polyommatus amorata* | TTATTAAATTTAA | 13 | 2 | - |
| *Agriades orbitulus* NQ2 | AAAATTAATAATTA | 14 | 2 | - |
| *Agriades orbitulus* NQ1 | AAAATTAATAATTA | 14 | 2 | 1 |
| *Agriades orbitulus* MY | AAAATTAATAATTA | 14 | 2 | 1 |
