# Supplementary material for: Comparative mitogenomic and evolutionary analysis of Lycaenidae (Insecta: Lepidoptera): Potential association with high-altitude adaptation
Source: Front Genet. 2023 Apr 18;14:1137588. doi: 10.3389/fgene.2023.1137588 (PMC10151513; doi:10.3389/fgene.2023.1137588)
Supplement: Supplementary file 1 [file DataSheet1.ZIP › Supplemental Materials Revised/Table S7 Estimation of a╪ values for the 13 mitochondrial protein-coding genes.docx]

**Table S7** Estimation of *ω* (*d*_N_/*d*_S_) values for the 13 mitochondrial protein-coding genes. 2Δ*ℓ*, twice the absolute value of likelihood of different models.

| Gene | Estimated *ω* values | | 2Δ*ℓ* | *P* value |
| --- | --- | --- | --- | --- |
|  | Foreground | Background |  |  |
| *atp6* | 0.0222 | 0.0346 | 0.98 | 0.323 |
| *atp8* | 0.1647 | 0.0108 | 5.35 | 0.021 |
| *cob* | 0.0184 | 0.0130 | 0.65 | 0.420 |
| *cox1* | 0.0086 | 0.0041 | 2.99 | 0.084 |
| *cox2* | 0.0171 | 0.0076 | 1.96 | 0.161 |
| *cox3* | 0.0178 | 0.0112 | 1.10 | 0.295 |
| *nad1* | 0.0169 | 0.0330 | 2.62 | 0.106 |
| *nad2* | 0.0163 | 0.0182 | 0.06 | 0.806 |
| *nad3* | 0.0056 | 0.0078 | 0.19 | 0.666 |
| *nad4* | 0.0169 | 0.0153 | 0.07 | 0.794 |
| *nad4L* | 0.0099 | 0.0017 | 3.69 | 0.055 |
| *nad5* | 0.0171 | 0.0207 | 0.34 | 0.559 |
| *nad6* | 0.0374 | 0.0060 | 5.79 | 0.016 |
