# Supplementary material for: Comparative mitogenomic and evolutionary analysis of Lycaenidae (Insecta: Lepidoptera): Potential association with high-altitude adaptation
Source: Front Genet. 2023 Apr 18;14:1137588. doi: 10.3389/fgene.2023.1137588 (PMC10151513; doi:10.3389/fgene.2023.1137588)
Supplement: Supplementary file 1 [file DataSheet1.ZIP › Supplemental Materials Revised/Table S8 Positively selective sites.docx]

**Table S8** Positively selected sites in *cox1* when the ancestral clade of two Polyommatinae species (*Polyommatus amorata* and *Agriades orbitulus*) inhabiting the Qinghai-Tibetan Plateau as the foreground branch.

| Model | Likelihood (*ℓ*) | Site class | 0 | 1 | 2a | 2b | 2Δ*ℓ* | *P* value | Positively selected sites |
| --- | --- | --- | --- | --- | --- | --- | --- | --- | --- |
| Model A (Null) | -2751.929629 | proportion | 0.9892 | 0.0048 | 0.0060 | 0.0000 |  |  | 404N 0.962* |
|  |  | background ω | 0.0041 | 1.0000 | 0.0041 | 1.0000 |  |  |  |
|  |  | Foreground ω | 0.0041 | 1.0000 | 1.0000 | 1.0000 |  |  |  |
| Model A (Alternative) | -2750.0895 | proportion | 0.9923 | 0.0047 | 0.0030 | 0.0000 |  |  |  |
|  |  | Background ω | 0.0041 | 1.0000 | 0.0041 | 1.0000 |  |  |  |
|  |  | Foreground ω | 0.0041 | 1.0000 | 11.1111 | 11.1111 | 3.6802 | 0.0550 |  |
