# Supplementary figures and images for: Comparative mitogenomic and evolutionary analysis of Lycaenidae (Insecta: Lepidoptera): Potential association with high-altitude adaptation
Source: Front Genet. 2023 Apr 18;14:1137588. doi: 10.3389/fgene.2023.1137588 (PMC10151513; doi:10.3389/fgene.2023.1137588)

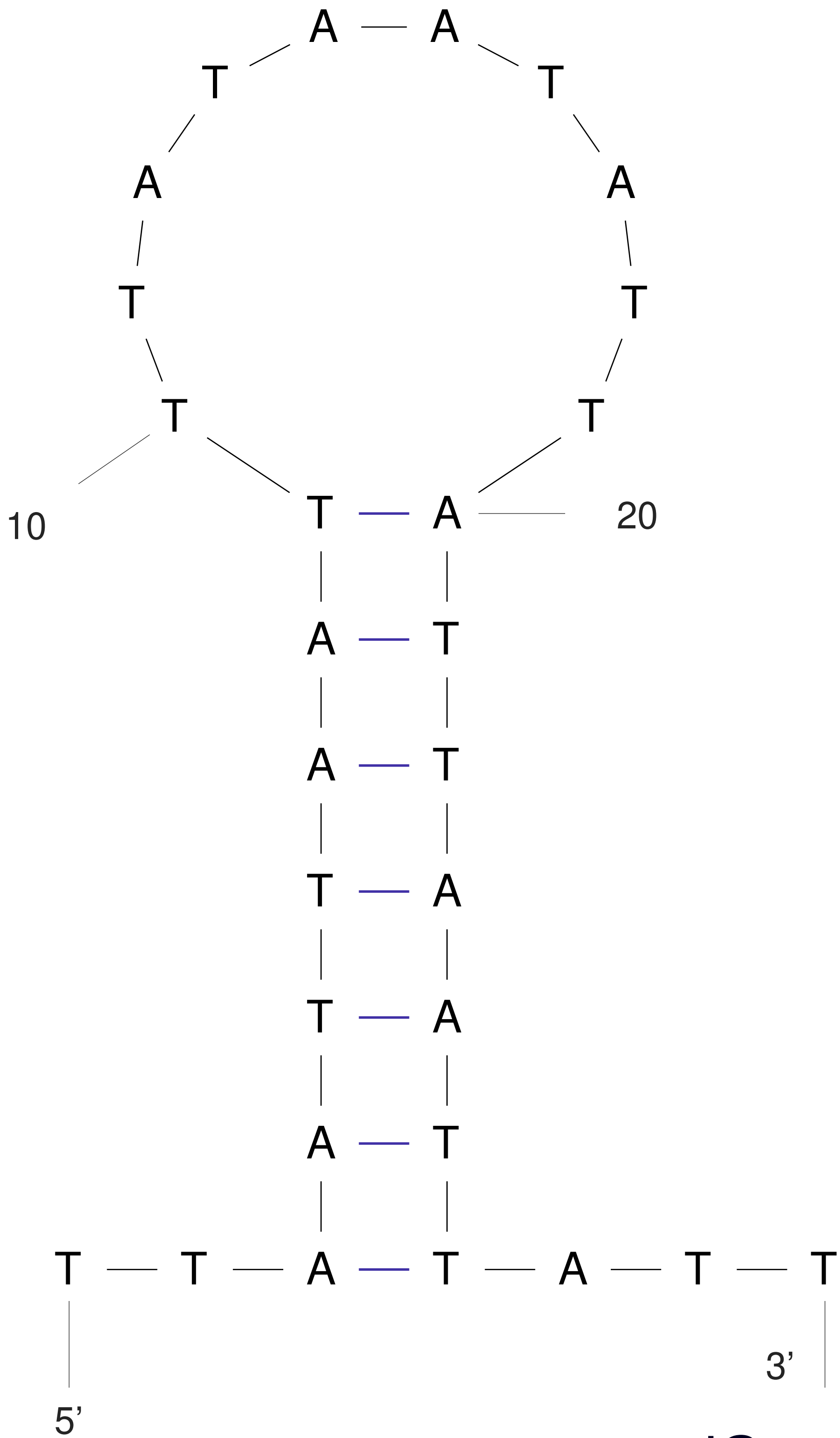

$$\Delta G = -2.48$$

Supplement: Supplementary file 1 [file DataSheet1.ZIP › Supplemental Materials Revised/Figure S2 Non-coding region between cox3 and trnG.pdf]

A. P123\_MrBayes

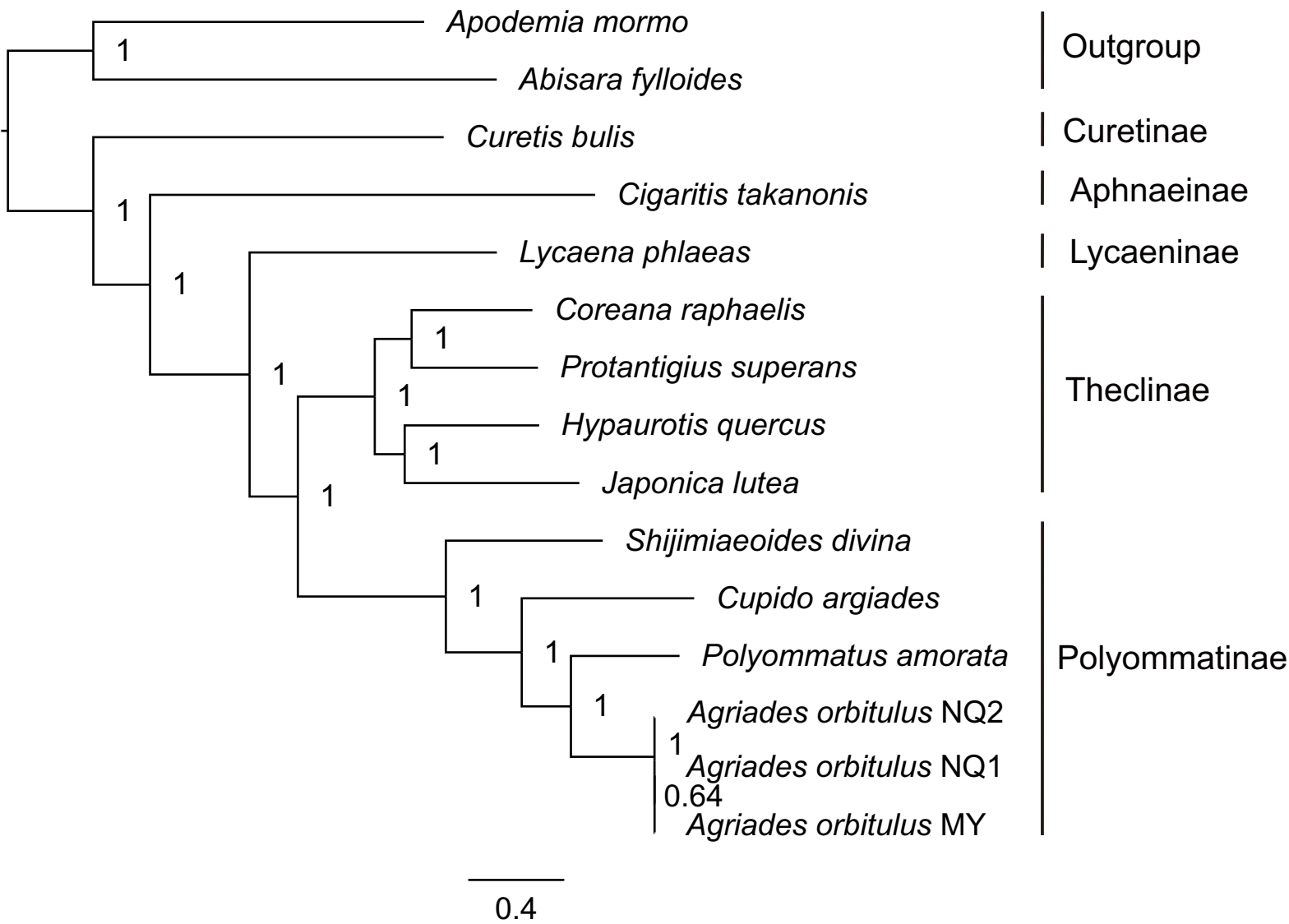

B. P123AA\_MrBayes

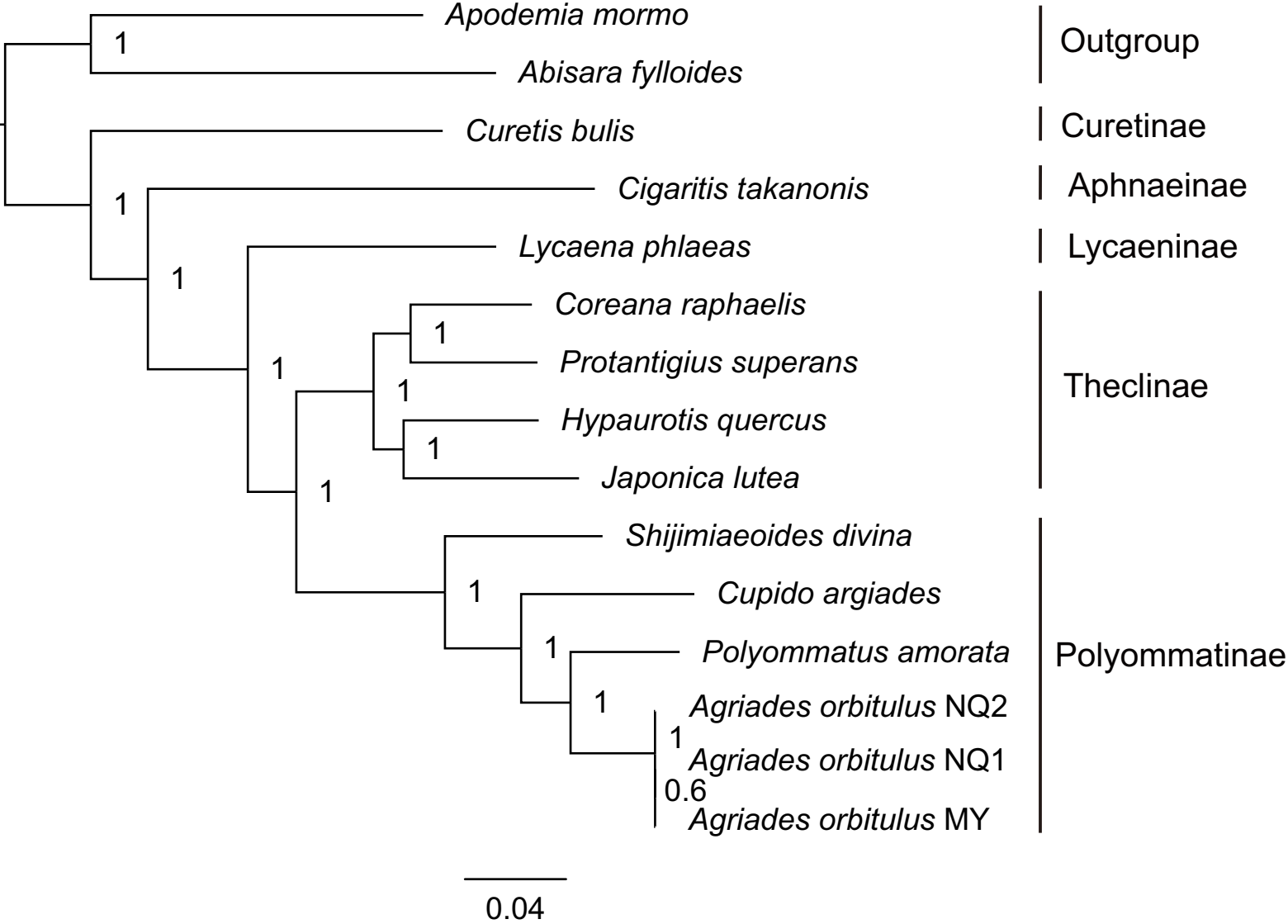

C. P123RNA\_MrBayes

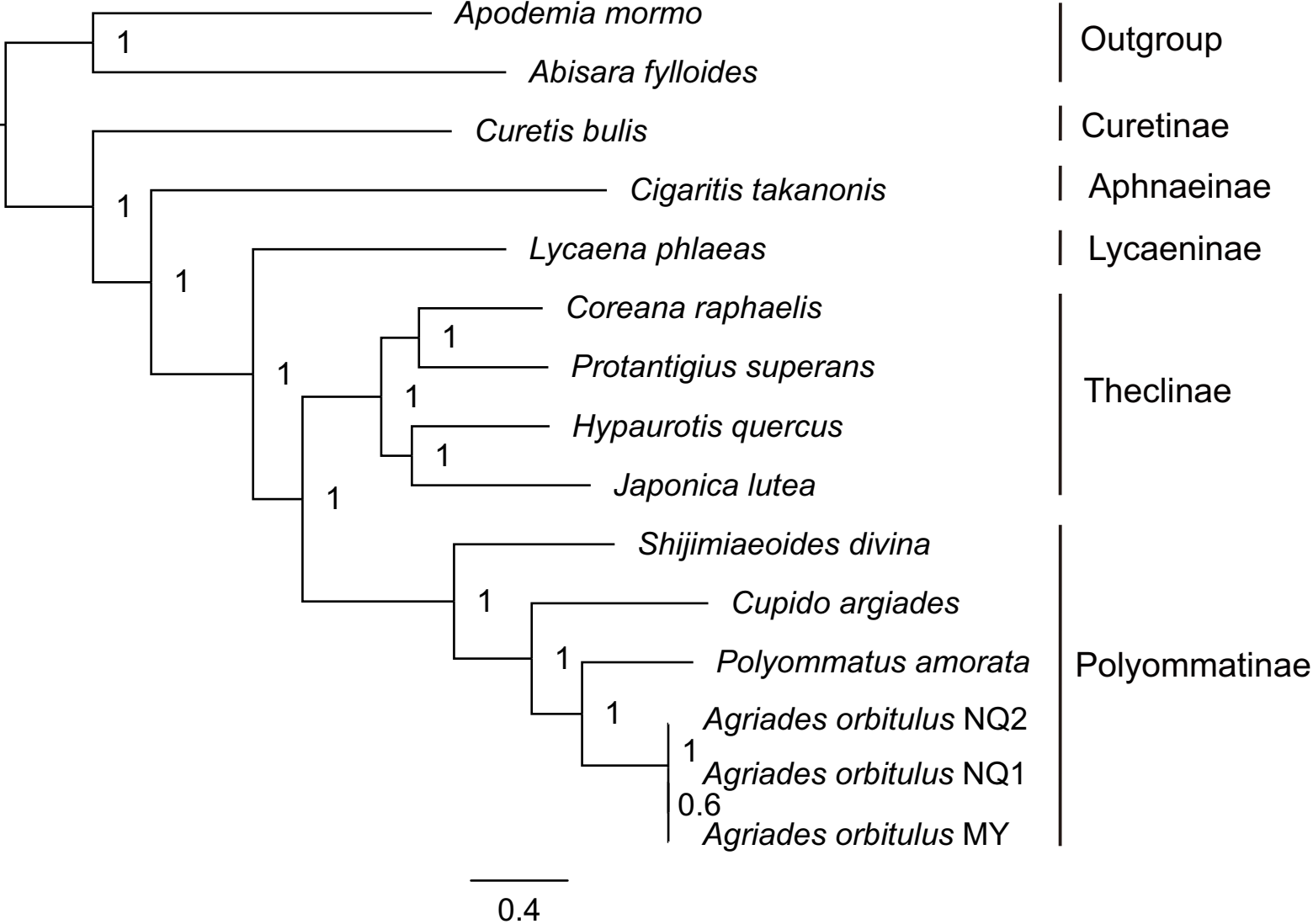

D. P123\_RAxML

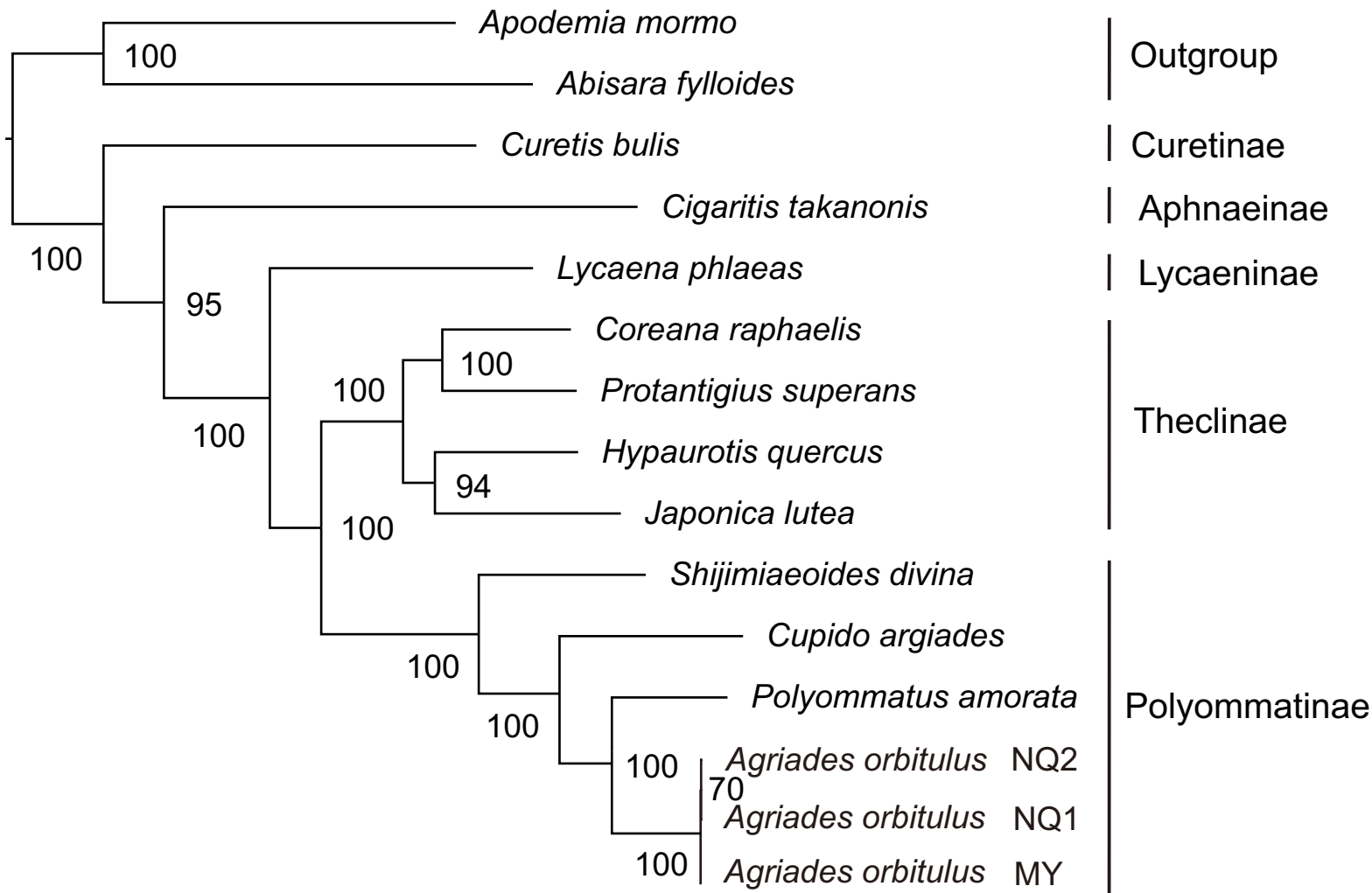

0.1

E. P123AA\_RAxML

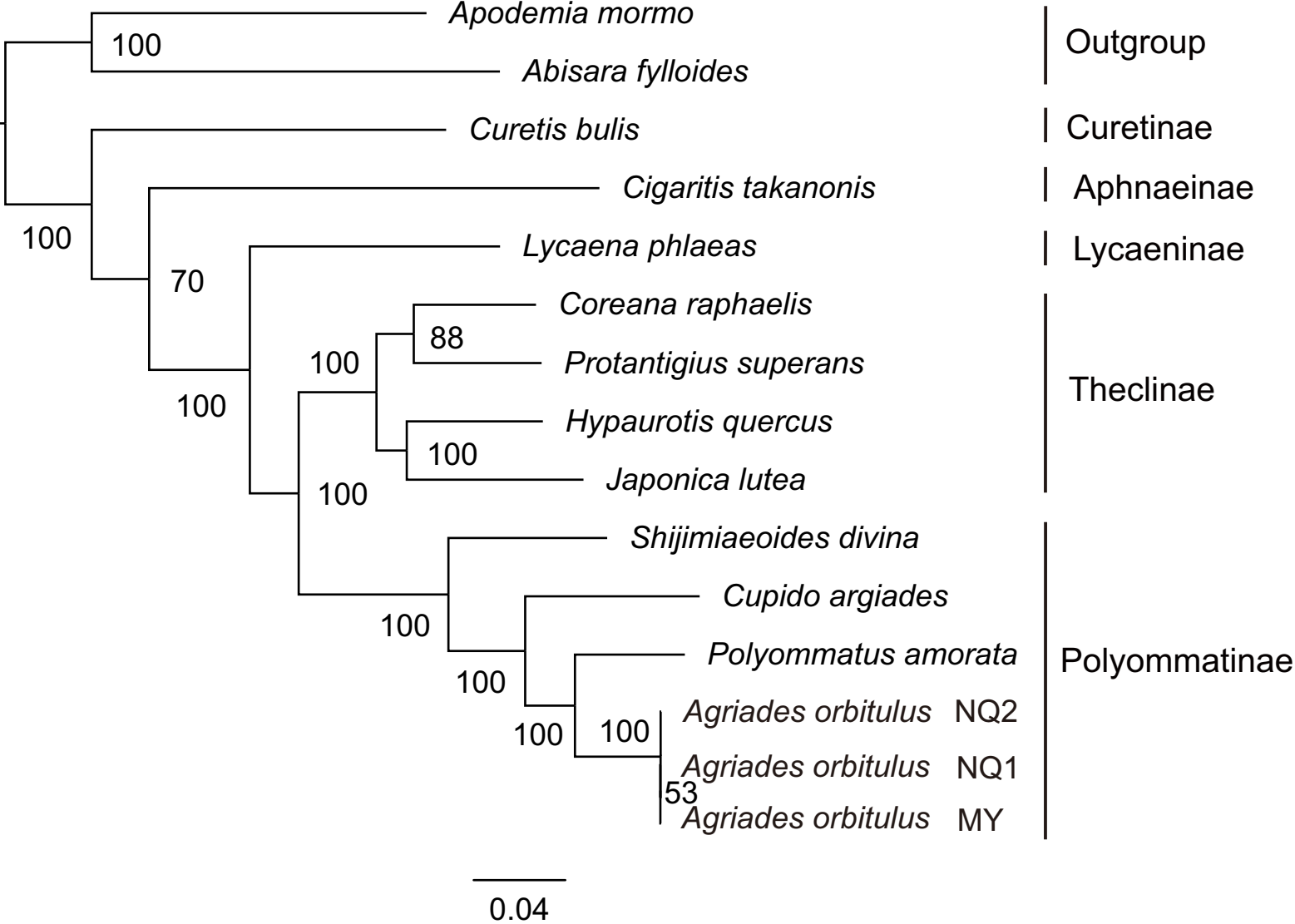

## F. P123RNA\_RAxML

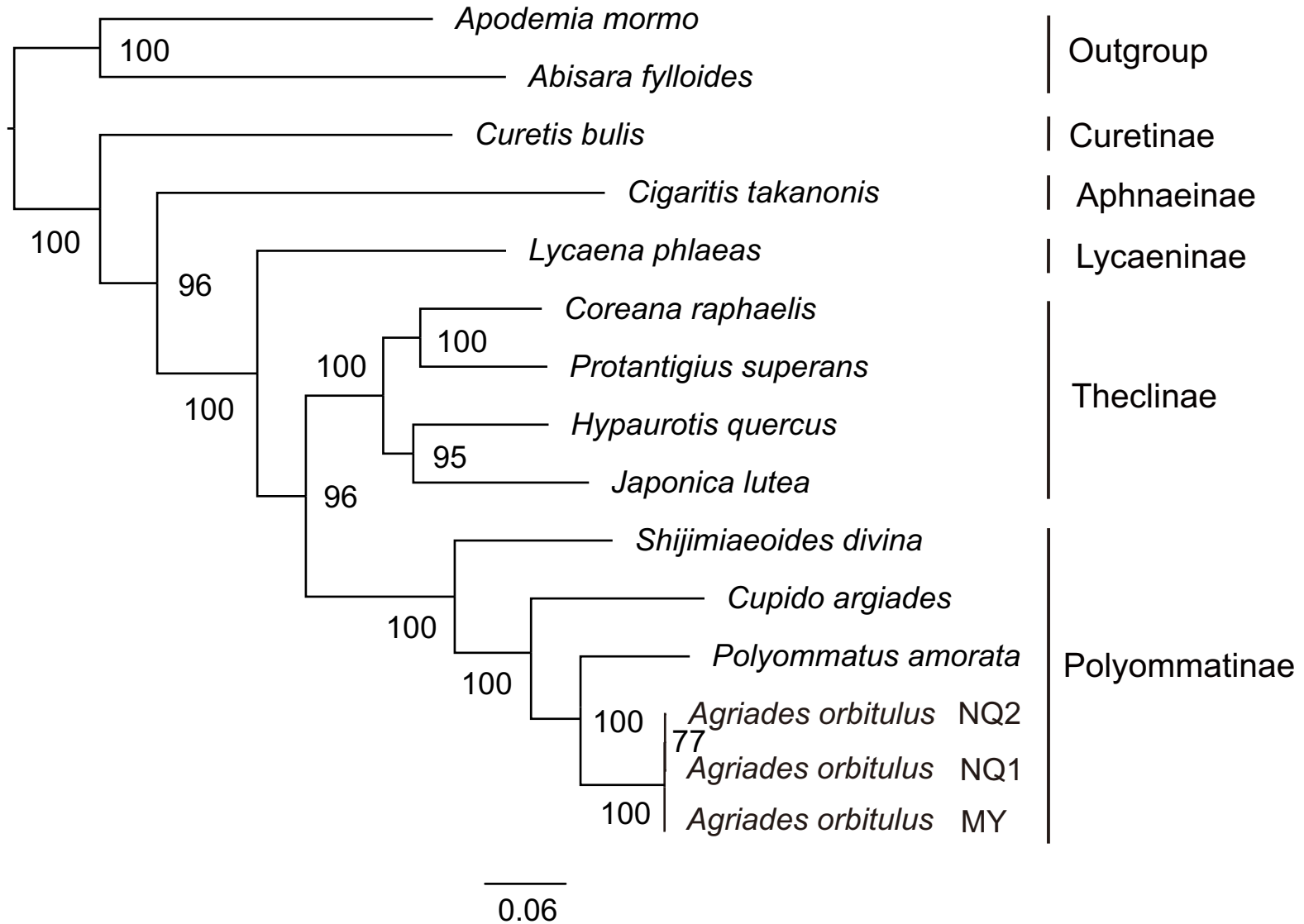

Supplement: Supplementary file 1 [file DataSheet1.ZIP › Supplemental Materials Revised/Figure S3 Phylogenetic tree.pdf]
